# Single-cell profiling of tumor heterogeneity and the microenvironment in advanced non-small cell lung cancer

**DOI:** 10.1038/s41467-021-22801-0

**Published:** 2021-05-05

**Authors:** Fengying Wu, Jue Fan, Yayi He, Anwen Xiong, Jia Yu, Yixin Li, Yan Zhang, Wencheng Zhao, Fei Zhou, Wei Li, Jie Zhang, Xiaosheng Zhang, Meng Qiao, Guanghui Gao, Shanhao Chen, Xiaoxia Chen, Xuefei Li, Likun Hou, Chunyan Wu, Chunxia Su, Shengxiang Ren, Margarete Odenthal, Reinhard Buettner, Nan Fang, Caicun Zhou

**Affiliations:** 1 Department of Medical Oncology, Shanghai Pulmonary Hospital, Tongji University School of Medicine, Shanghai, China; 2 Singleron Biotechnologies, Nanjing, Jiangsu China; 3 Department of Pathology, Shanghai Pulmonary Hospital, Tongji University School of Medicine, Shanghai, China; 4 Institute of Pathology, University Hospital of Cologne, Cologne, Germany; 5 Center for Molecular Medicine Cologne, University of Cologne, Cologne, Germany

**Keywords:** Cancer genomics, Cancer microenvironment, Non-small-cell lung cancer, Tumour heterogeneity

## Abstract

Lung cancer is a highly heterogeneous disease. Cancer cells and cells within the tumor microenvironment together determine disease progression, as well as response to or escape from treatment. To map the cell type-specific transcriptome landscape of cancer cells and their tumor microenvironment in advanced non-small cell lung cancer (NSCLC), we analyze 42 tissue biopsy samples from stage III/IV NSCLC patients by single cell RNA sequencing and present the large scale, single cell resolution profiles of advanced NSCLCs. In addition to cell types described in previous single cell studies of early stage lung cancer, we are able to identify rare cell types in tumors such as follicular dendritic cells and T helper 17 cells. Tumors from different patients display large heterogeneity in cellular composition, chromosomal structure, developmental trajectory, intercellular signaling network and phenotype dominance. Our study also reveals a correlation of tumor heterogeneity with tumor associated neutrophils, which might help to shed light on their function in NSCLC.

## Abstract (web summary)

Comprehensive profiles of tumour and microenvironment are critical to understand heterogeneity in non-small cell lung cancer (NSCLC). Here, the authors profile 42 late-stage NSCLC patients with single-cell RNA-seq, revealing immune landscapes that are associated with cancer subtype or heterogeneity.

## Introduction

Tumor ecosystems are comprised of cancer cells, infiltrating immune cells, stromal cells, and other cell types together with noncellular tissue components, which interact and collectively determine disease progression as well as response to therapy^1,2^. It is well known that cancer patients elicit very individualized responses to different treatments, demanding better characterization of the whole tumor ecosystem beyond currently applied clinical typing of somatic mutations in cancer cells. Furthermore, precisely targeted therapies against well-defined oncogenic drivers reveal a wide spectrum of responses in different settings. For example, the *KRAS* G12C inhibitors seemed to induce tumor responses in the majority of lung cancers but much less in pancreatic cancers, which differ in their tumor micromilieu dominated by cancer-associated fibroblasts^3^. Antibodies targeting PD1 or PD-L1 have achieved substantial overall survival improvements in advanced non-small cell lung cancer (NSCLC). The 5-year survival rate could be prolonged from less than 5–29.6% in PD-L1-positive patients^4,5^. However, major challenges still remain, including low response rate in unselected patients, lack of reliable predictive biomarkers, and identification of more immunotherapeutic targets. Thus, comprehensive understanding of NSCLC ecosystems holds the promise to improve personalized treatment strategies^6^.

Conventional ‘bulk’ RNA-sequencing methods process a mixture of all cells, averaging out underlying differences in cell-type-specific transcriptomes. In contrast, single-cell RNA-sequencing (scRNA-seq) profiles the gene expression pattern of each individual cell and decodes its intercellular signaling networks. This unbiased characterization provides clear insights into the entire tumor ecosystem, such as mechanisms of intratumoral and intertumoral heterogeneity, as well as cell–cell interactions through ligand-receptor signaling^7^. Thus, several studies deeply characterized the lung tumor microenvironment (TME) at single-cell resolution. An extensive taxonomy of stromal cells with different pathway activities in NSCLC patients presented a first-ever lung cancer TME cell atlas^8^. Isolated infiltrating T cells in NSCLC were classified according to their functional states and dynamics and a subset of regulatory T cells (Tregs) was found to correlate with the poor prognosis in lung adenocarcinoma^9^. Tumor-infiltrating myeloid cells (TIMs), including monocyte, macrophage, dendritic, and granulocyte cell lineages, were categorized into at least 25 different states by scRNA-seq^10^. Subsets of TIMs defined by unique markers have been associated with patient prognosis. Heterogeneity of tumor endothelial cells was also studied for both human and mouse^11^. All reports mentioned above focused on early stage, resectable lung cancers, which may not reflect the cellular profiles of tumors at advanced stages that have undergone intense and exhaustive interactions with stromal and immune cells. Focusing on the evolutional dynamics of lung adenocarcinoma, Kim’s study was performed on the lung adenocarcinoma samples from early-stage tissues to advanced stage biopsies including both primary and metastatic sites^12^. Another recent study uncovered transcriptional signatures specific to various targeted therapies and clinical states on primary and metastatic lung biopsies by low throughput Smart-seq2 technology, which only included one squamous carcinoma patient^13^. Until now, the late-stage landscape of lung squamous carcinoma was mostly absent.

In this study, we apply scRNA-seq to analyze the cancer and TME landscape of advanced NSCLC for both lung adenocarcinoma and squamous carcinoma. We identify distinct cell populations and cellular signals that are differentially enriched in tumors depending on the pathological types, presence or absence of driver mutations, and degree of tumor heterogeneity. Our data provide a comprehensive scRNA-seq profiling on a large number of small biopsies and may be used to improve diagnostics and prognosis in clinical settings.

## Results

### Establishment of advanced NSCLC cell atlas

**Fig. 1:**
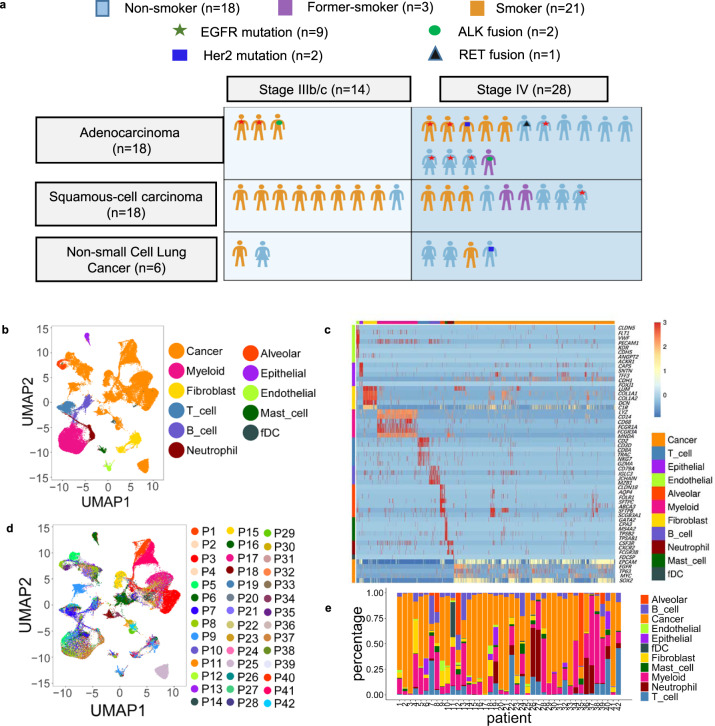
Advanced NSCLC single-cell atlas. **a** Graph illustration of the baseline information of the 42 patients, including subtypes, stages, mutation status, and smoking history. **b** UMAP plot of 90,406 cells from 42 patients, colored by their 11 major cell types. **c** Heatmap of canonical cell-type markers of 11 major cell types. **d** UMAP plot of all cells, colored by patients. **e** Major cell-type composition of each patient. Biopsies were all taken from the primary lung tumors. Source data are provided as a Source Data file.

We applied scRNA-seq analyses to biopsy samples from 42 advanced NSCLC patients with diverse histological and molecular phenotypes and treatment history (Fig. 1a, Supplementary Table 1). Following multiple quality control and filtering steps, a total of 90,406 cells were analyzed with respect to their transcriptomes. By characteristic canonical cell markers, eleven major cell types were detected, classified as carcinoma cell types, epithelial cells others than carcinoma cells, immune cell types (T cells, B lymphocytes, myeloid cells, neutrophils, mast cells, and follicular dendritic cells) and stromal cell types (fibroblasts and endothelial cells) (Fig. 1b, c and Fig. S1). Similar to the observations in previous studies, stromal and immune cells of different patients clustered together by cell types, while cancer cells showed higher heterogeneity and patient-specific expression signatures (Fig. 1d)^14,15^. Similar to the observations from previous studies^8,12^, the portions of cancer, stromal, and immune cells varied greatly among samples, which could be intrinsic to different tumor phenotypes or related to locations within the tumor where biopsies were taken (Fig. 1e and Supplementary Data 1). For example, tumor specimen P42 (lung adenocarcinoma mixed with sarcomatoid carcinoma) and P7 revealed a strongly inflammatory micromilieu with almost 50% T cells in contrast to specimen P2, P3, and P17, which were practically T cell depleted.

### Lung Squamous Carcinoma has higher inter- and intratumor heterogeneity than lung adenocarcinoma

**Fig. 2:**
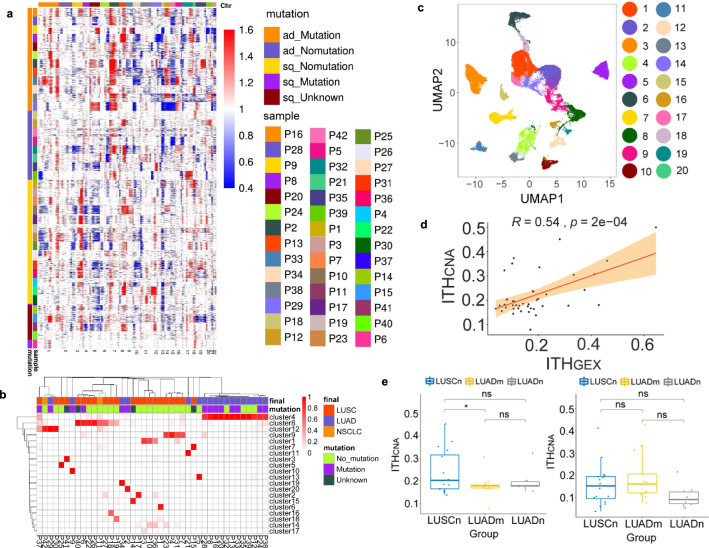
Inter- and intratumor heterogeneity of cancer cells. **a** Heatmap of CNA profiles inferred from scRNA-seq of tumor cells of patients. Red indicated genomic amplifications and blue indicated genomic deletions. The *x*-axis showed all chromosomes in the numerical order. The *y*-axis was marked by both patient subgroups. **b** Heatmap displaying proportions of cancer cells of each patient in cancer clusters. The clustering results of cancer cells were generated using resolution 0.4 in Seurat. The arrangement of the patients on the *y*-axis were based on their similarities using hierarchical clustering. **c** UMAP visualization of cancer cell clusters. The cluster IDs corresponded to cluster IDs shown in **b**. **d** Correlation between ITH_CNA_ and ITH_GEX_ for 42 patients. Shaded areas corresponded to the 0.95 confidence interval analyzed by two-sided *t*-test. **e** Statistical tests of ITH_CNA_ and ITH_GEX_ between patients in different groups, LUSCn, LUADm and LUADn (LUSCn: *n* = 16, LUADn: *n* = 6, LUADm: *n* = 12 biological independent samples. *p ≤ 0.05; ns: *p* > 0.05). Two-sided unpaired Wilcoxon test was performed to compare between groups. The lower hinge, middle line, and upper hinger of boxplots represented the first, second, and third quartiles of the distributions. The upper and lower whiskers corresponded to the largest and smallest data points within the 1.5 interquartile range. All actual data values were also plotted as dots alongside the boxplots.

Based on single-cell expression levels of genes commonly used as markers for immunohistochemistry-based NSCLC classification, namely *NAPSA*, TTF-1 (*NKX2-1*) for lung adenocarcinoma (LUAD), and *TP63*, CK5 (*KRT5*) for lung squamous carcinoma (LUSC), subtype classifications aligned well with the histopathological classifications (Fig. S2). Next, we used the scRNA-seq data to infer copy number alterations (CNAs) in cancer cell populations. The inferred CNA profiles of 42 patients showed both interpatient and intrapatient heterogeneity (Fig. 2a). For LUAD patients, prominent arm-level insertions were found in chromosome 7 and 8q, with deletions in chromosome 10. Noteworthy, LUAD with known driver mutations have additional amplifications in the 1q and 5p arms. In contrast, LUSC patients mostly have 3q insertions and 5q deletions. Interestingly, some of the LUAD patients without driver mutations have similar CNA profiles to LUSC. Although expression profiles and composition of the cancer cell transcriptomes were largely patient-specific, carcinoma cells from some patients were more similar than others (Fig. 2b and Fig. S3A, B). In most cases, cancer cells from LUAD and LUSC patients partitioned into separate clusters. More than half of the LUAD patients clustered into one group, while most LUSC tumors formed patient-specific clusters, indicating higher intertumor differences in LUSC than in LUAD. Most patients, especially patients with LUAD e.g., P16, P20, and P32, had dominant clones, while in a few LUSC such as P27 and P37 the malignant cells spread across multiple clusters (Fig. 2b and Fig. S3C). LUSC patients showed significantly higher clonality than LUAD patients (Fig. S3D).

To quantify the intratumoral heterogeneity, we defined both a CNA-based and an expression-based intratumor heterogeneity score, denoted as ITH_CNA_ and ITH_GEX_ (see Methods for their definitions). We observed various degrees of heterogeneity within the tumor (Fig. S4A, B). ITH_CNA_ and ITH_GEX_ showed a moderate correlation (Fig. 2d), potentially due to the nondriver genomic alternations or the microenvironment shaped tumor phenotypes. We further divided patients into three groups according to both the cancer type and mutation: LUAD patients with driver mutation (*n* = 12), denoted as LUADm, LUAD patients without driver mutation (*n* = 6), denoted as LUADn, and LUSC patients without driver mutation (*n* = 16), denoted as LUSCn. Interestingly, LUSCn patients have significant higher ITH_CNA_ comparing to patients of LUADm, while there was no statistical significance in terms of ITH_GEX_ (Fig. 2e). This finding also suggested patients with driver mutations may be phenotypically influenced beyond genomic alternations. The comparison between this cohort and a cohort from public data revealed increased ITH_GEX_ scores of late-stage patients^12^ (Fig. S4C).

### Plasticity of lung epithelial cells and their developmental trajectories into malignant tumor cells

**Fig. 3:**
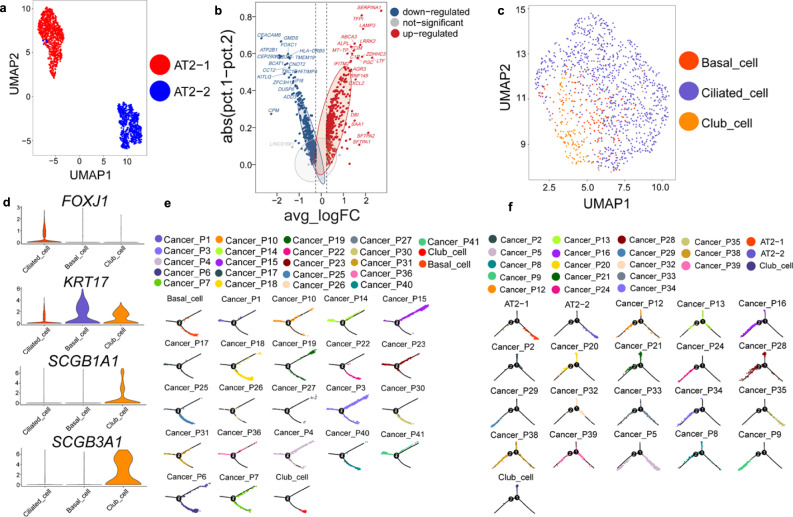
Phenotypes of lung epithelial cells and their evolutionary trajectory into cancer cells. **a** UMAP projection of alveolar cells. Alveolar cells could be further divided into two clusters, both of which are AT2 cells. They were denoted as AT2-1 and AT2-2. **b** Volcano plot of differentially expressed genes between AT2-1 and AT2-2 cells. Difference between percentage of cells expressed in two clusters was plotted against log fold change of average expressions. **c** UMAP visualization of epithelial cell subtypes. Epithelial cells could be further annotated into basal cells, club cells, and ciliated cells. **d** Heatmap of canonical marker genes of epithelial lung subtypes. **e** Developmental trajectories of AT2 cells, club cells, and LUAD tumor cells. Normal cells were shown as a whole for each type, and cancer cells were shown separately for each patient. **f** Developmental trajectories of basal cells, club cells, and LUSC tumor cells.

All identified alveolar cells express the canonical markers (*CLDN18*, *SFTPA1*, *SFTPC*) of Alveolar Type 2 cells (AT2) without expressing Alveolar Type 1 cell markers (*CAV1*, *AGER*). Further clustering analysis unveiled two distinct cluster of AT2 cells, denoted as AT2-1 and AT2-2 (Fig. 3a). AT2-2 resembled a normal AT2 phenotype with common AT2 markers *SFTPA* and transporter *ABCA3* upregulated (Fig. 3b). In contrast, AT2-1 expressed cell proliferation and cell migration related genes, such as *CEACAM6*, *KITLG*, and *FOXC1*, implying a phenotypic change towards malignancy. Epithelial cells could be further separated into ciliated epithelial cells, club cells, and basal cells (Fig. 3c, d).

Previous studies showed that AT2 cells and club cells could both develop into LUAD cells, while basal cells and club cells are potential progenitors of LUSC^16,17^. Therefore, we organized AT2 cells, club cells and LUAD cancer cells according to their developmental trajectories (Fig. 3e). The inferred pseudotime paths showed AT2 cells and club cells transited into LUAD tumors independently. In contrast, basal cells seemed to act as a transitional state between club cells and LUSC tumor cells (Fig. 3f). Besides such distinct signatures, we found tumor cells of some patients clustered closely at the end of the branches, implying a homogeneous and terminal phenotype, while others have more diverse and heterogeneous profiles spreading along cancer developmental trajectories.

### Advanced NSCLC TME revealed a rich program of stromal and immune components

To further identify subgroups of each stromal and immune cell types, we clustered and annotated them individually. We identified five subtypes of endothelial cells (EC) including lymphatic, venous, and arterial endothelial cells (LEC, VECs, and AECs), tip cells, and an EC cluster enriched with interferon induced genes (Fig. S5 and Supplementary Data 2). Furthermore, we divided fibroblasts into pericytes and fibroblasts, including six subclusters of fibroblasts (Fig. S6 and Supplementary Data 3). For immune cells, our data revealed two B cell subgroups and seven different plasma cells (Fig. S7). Myeloid cells, especially macrophages, have a broad range of phenotypes and could be divided into 10 different groups (Fig. S8). Dendritic cells (DCs), including plasmacytoid dendritic cells (pDCs), conventional type 1 and 2 DCs (cDC1 and cDC2), and mature DCs were also discovered. Neutrophils have two distinct clusters, expressing potential polymorphonuclear myeloid-derived suppressor cells (PMN-MDSCs) related genes such as LOX-1 (*OLR1*) to different extents (Fig. S9).

### Detailed analysis of T cells uncovered Th17-like cells and their potential interconversion with Tregs

**Fig. 4:**
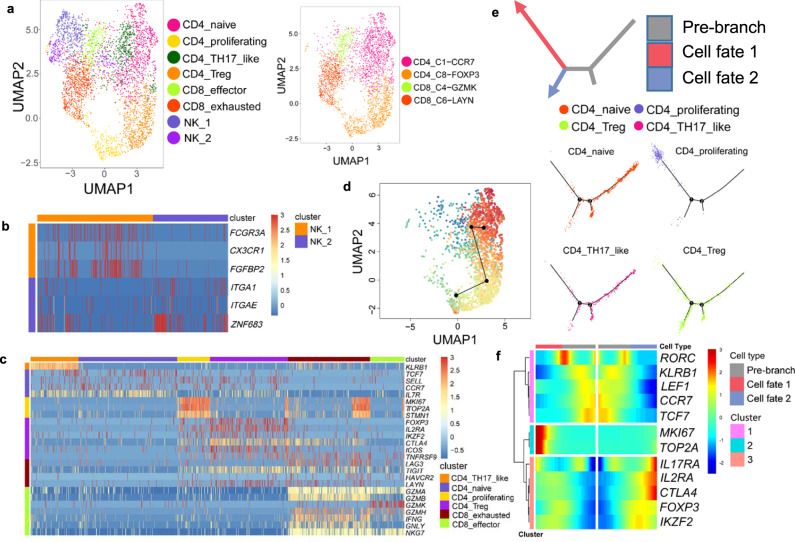
Subtypes and developmental trajectory of T cells. **a** UMAP visualization of 6 T cell subtypes and 2 NK cell subtypes (left) and predicted T cell subtypes by singleR (right). **b** Heatmap of selected markers for 2 NK clusters. **c** Heatmap of T subtype markers and selected functional genes. **d** Transitional relationship among CD4 T cells predicted by Slingshot. Rainbow coloring from red to blue represented the begin to end of the trajectory. **e** Illustration of CD4 T cell differentiation pathways inferred by Monocle and the relative locations of each CD4 T subtypes along the development pathways. The red and blue arrows indicated the two pseudotime directions of cell development. The grey section represented the beginning of the trajectory before the branching point. **f** Heatmap showing relative expressions of canonical markers of CD4 T cells along inferred trajectories. The red and blue branches correspond to the two developmental directions in **e**.

Within tumor-infiltrating T cells, we identified CD4+ naïve T cells, CD4+ Tregs, CD4+ T helper 17-like T cells (Th17-like), CD8+ effector T cells, CD8+ exhausted T cells, and Natural Killer (NK) cells according to expression of their respective markers (Fig. 4a). T cell subtypes were confirmed by supervised cell-type annotation based on previously studied T subtype expression profiles^9^ (Fig. 4a). To further characterize two NK clusters (*CD3D*−, *KLRD1*+, *NKG7*+), we referred to the CD16+ (*FCGR3A*) cluster as NK-1 and CD16− cluster as NK-2 (Fig. 4b). NK-1 contains upregulated transcripts encoding fractalkine receptor (*CX3CR1*) and fibroblast growth factor binding protein 2 (*FGFBP2*), both involved in lymphocyte cytotoxic functions. NK-2 had higher expression of tissue-resident markers such as CD49a (*ITGA1*), CD103 (*ITGAE*), and *ZNF683*. Co-inhibitory immune checkpoints including *CTLA4* and *TIGIT* were enriched in CD4+ Tregs and CD8+ exhausted T cells (Fig. 4c). However, *LAG3* was mainly expressed in CD8^+^ exhausted T cells, which is consistent with previous findings^9^.

We then performed trajectory analysis on CD4^+^ T cells to determine their developmental pathways within TME using both Slingshot^18^ and Monocle^19^. Slingshot revealed a transitional relationship between Tregs and Th17-like cells, originated from naïve cells (Fig. 4d). Uncovered by Monocle, naïve cells differentiated into two major branches, Tregs and a proliferating population (Fig. 4e, f). Interestingly, Th17-like cells, confirmed by expression of their master transcription factor *RORC*, showed a transitional phenotype spreading along the developmental pathway from naïve cells to Tregs (Fig. 4f). The CD4^+^ Th17-like population marked by high expression of gene *KLRB1*^20^ is, to our knowledge, the first report of Th17-like cells identified in NSCLC tumor environments by scRNA-seq. As supported by literature^21^, natural Tregs (nTregs), a subset of Tregs, are believed to interconvert with Th17-like cells. This result revealed a complex and delicate interplay between Tregs and Th17-like cells and highlighted the importance of their balance in adaptive immune responses to tumor antigens^22^.

### Both NSCLC subtypes and ITH shaped the immune landscape in TME

**Fig. 5:**
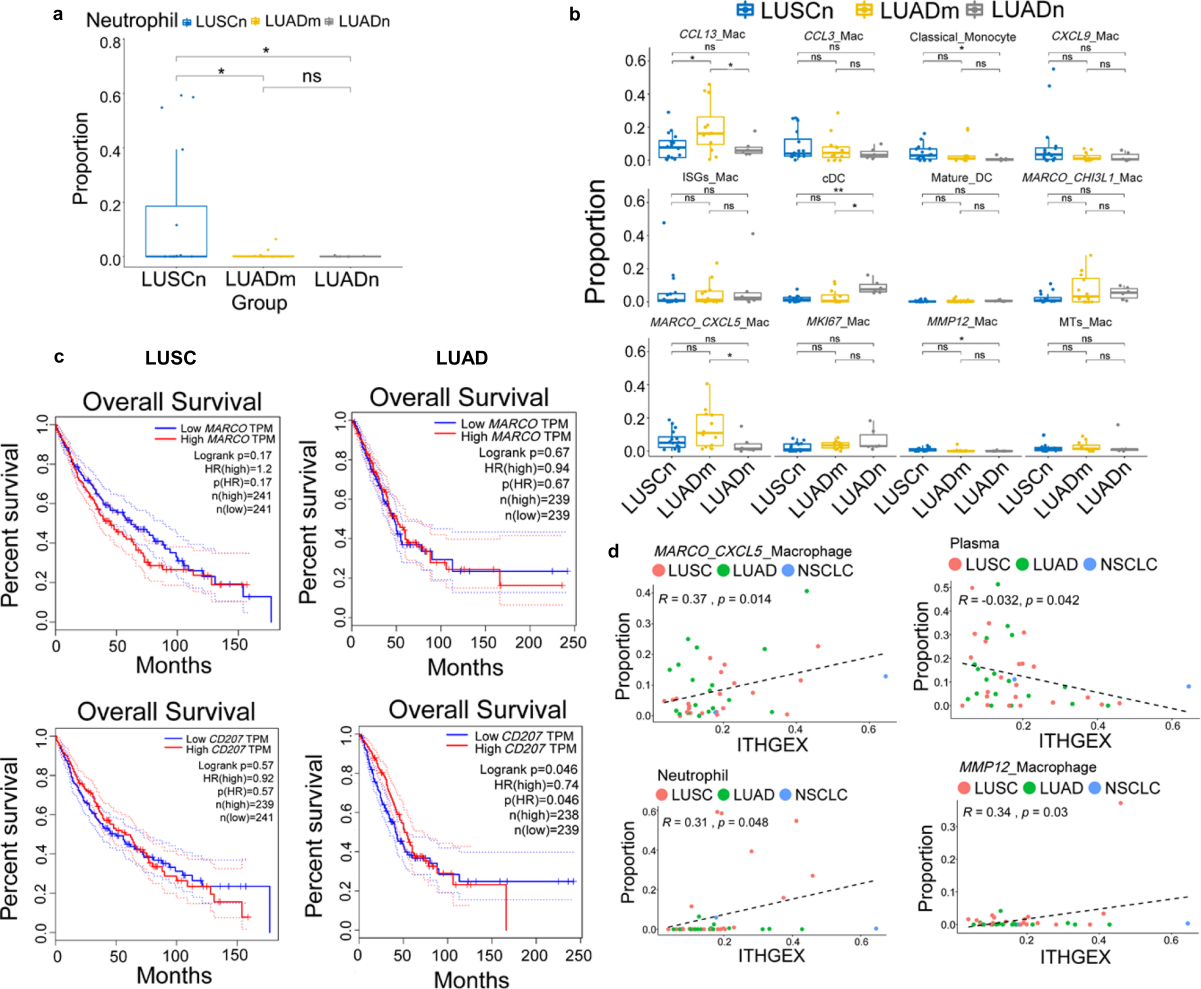
Correlation analysis of cellular composition, tumor subtypes, and ITH. Cellular composition analysis of cell type between patient groups for **a** neutrophils and **b** macrophage subtypes. Two-sided unpaired Wilcoxon test was performed to compare between groups for tests in **a** and **b** (LUSCn: *n* = 16, LUADn: *n* = 6, LUADm: *n* = 12 for both **a** and **b**. **p ≤ 0.01; *p ≤ 0.05; ns: *p* > 0.05). The lower hinge, middle line, and upper hinger of boxplots represented the first, second, and third quartiles of the distributions. The upper and lower whiskers corresponded to the largest and smallest data points within the 1.5 interquartile range. All actual data values were also plotted as dots alongside the boxplots. **c** Survival analysis for tissue-resident macrophage markers (*MARCO* and *CD207*) of LUAD and LUSC. **d** Correlation analysis between ITH_GEX_ and the cellular composition of patients. Only significantly associated cell types were shown. The tumor subtypes (LUAD, LUSC, and NSCLC) were shown in different colors and *p*-values were obtained by two-side *t*-tests (LUAD: *n* = 18, LUSC: *n* = 22, and NSCLC: *n* = 2).

To investigate if tumor subtypes and their ITH levels affect their microenvironment, we compared the cell-type composition of NSCLC by their histology and their driver mutation status. We found that neutrophils were significantly depleted in all LUAD patients (Fig. 5a). While comparing LUAD patients with and without oncogenic driver mutations, a macrophage cluster with highly expressed *CCL13* was enriched in the group of mutated tumors (Fig. 5b). The proportions of the tissue-resident macrophage cluster expressing the scavenger receptor *MARCO* and *CXCL5*, and cDCs also exhibited significant differences among the three groups (Fig. 5b). Interestingly, cDC2 displayed Langerin (*CD207*) expression, which was inferred to be dictated by the environment^23^. TCGA survival analysis revealed that *CD207* is a prognostic marker for LUAD, but not for LUSC (Fig. 5c). However, *MARCO* is not associated with clinical outcomes of both LUSC and LUAD. Since we identified two subtypes of *MARCO*+ alveolar macrophages, these results combined implied the multifunctional roles of tissue-resident myeloid cells. We next investigated the correlation between ITH scores and the immune cell compositions. We found neutrophils and two subtypes of macrophages were positively correlated with ITH_GEX_, while plasma cells were negatively correlated with ITH_GEX_ (Fig. 5d). This finding potentially suggested high immunosuppressive environment and low cancer killing ability for patients with high ITH_GEX_. Overall, we found the myeloid compartment is the mostly affected by tumor subtypes and ITH levels instead of tumor-infiltrating lymphocytes.

### Divergent intercellular networks observed among LUADn, LUADm, and LUSC

**Fig. 6:**
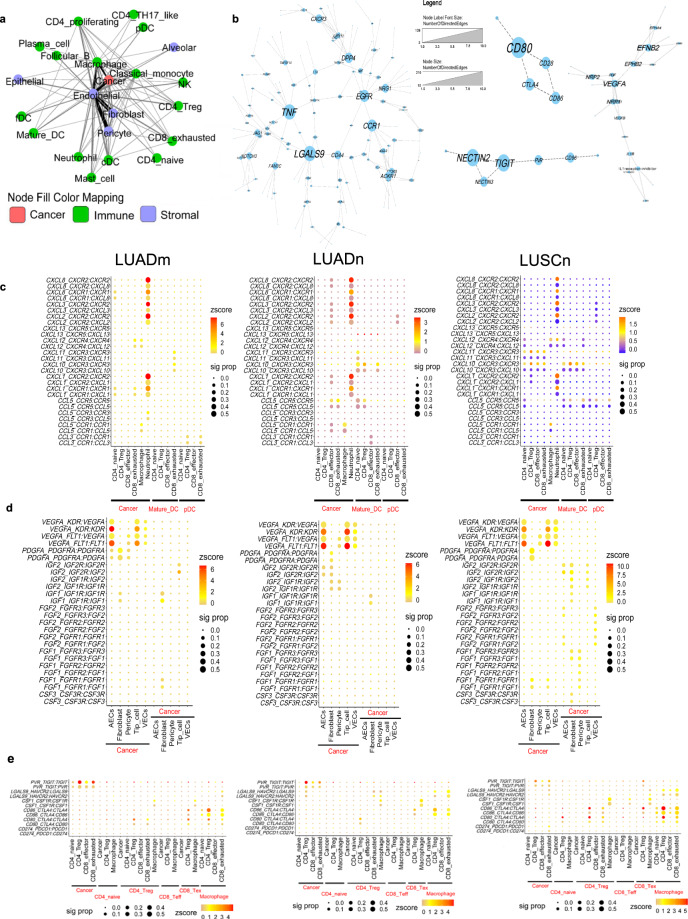
Cell and gene interaction networks. **a** The cellular interaction network among cell types of NSCLC patients. The line width and color were proportional to numbers of interactions between cell types. **b** Interacting molecular networks. Within each connected network, node (gene) sizes were proportional to the number of neighbors (interacting genes) of each node. Heatmaps shown selected interacting pairs for selected cell types in LUADm, LUADn, and LUSCn groups. Z-scores of expression levels were represented by color, and dot size displayed the proportion of patients who have significant interaction for the given ligand-receptor pair. **c** chemokine and chemokine receptors between cancer cells, T cells and DCs. **d** selected growth factors between cancer cells and stromal cells. **e** selected checkpoints between cancer cells, macrophages, and T cells.

In order to explore the interplay among cell types within the tumor microenvironment, we performed a cell–cell interaction analysis and showed a prominent interaction between cancer cells and endothelial cells, fibroblasts and macrophages (Fig. 6a). Analysis of the interacting molecules across cells showed a complex network with the interplay of oncogenic pathways as EGFR, NOTCH, WNT, with PDGF and inflammatory signaling pathways, in particular affecting TNF-a and chemokine responsive pathways (Fig. 6b). Notably, VEGFA-mediated protein–protein interactions and two analogous immune checkpoint pathways CD226-TIGIT-CD96 and CD274 (PD-L1)-CTLA4-CD28 were also identified within the interaction network.

Cancer cells expressed high levels of ligands *CXCL1*, *CXCL2*, *CXCL3*, and CXCL8, signaling to the receptors *CXCR1* and *CXCR2* expressed by neutrophils (Fig. 6c). Some of the LUSCn and LUADn patients showed increased interactions between DCs and T cells, including CXCR3 and its partners, suggesting strong effector T cell trafficking and recruiting^24^. We also confirmed activation of the CXCL12-CXCR4 pathway between tumor and sprouting endothelial cells (AECs and tip cells) described in Fig. S5. Regarding growth factors, the majority of tumors, regardless of subtypes, had very strong signals of VEGF interactions between tumor and various types of endothelial cells (Fig. 6d). PDGF signaling, on the other hand, was activated between tumor and cancer-associated fibroblast (CAF) cells. A distinct pattern for LUSC patients is the activation of FGF pathways among stromal cells and tumor cells, also supported by previous studies^25^. Since only a portion of LUSC patients have FGF pathways activated, patient stratification may be important for the usage of drugs targeting FGF pathways.

For patients’ immune environment, we found macrophages, instead of cancer cells, played a major role in inhibiting T cell functions through checkpoint pathways (Fig. 6e). Different subgroups showed different dominant pathways. For example, LUADm have high levels of activation of the TIGIT pathway but low levels of activation of TIM3 (*HAVCR2*) pathway. We did not detect any significant activation of the PD1/PD-L1 axis except for a few LUSC patients, potentially due to the low expression of PD1/PD-L1 on the transcriptomic level. Interaction analysis performed on a public dataset confirmed the similar activation state of checkpoint pathways for late-stage LUAD (Fig. S10A). Interestingly, an early-stage LUAD patient in the same dataset showed opposite activation status of *TIGIT* and TIM3 with respect to the late-stage patient (Fig. S10B). Nevertheless, by cellular network analysis of the scRNA-seq data, we generated a comprehensive view of patients’ TME including angiogenesis, CAF activation, recruitment of immunosuppressive cells, T cell activation, and detailed activation profiles of checkpoint pathways. Therapeutics related interactions were heterogeneous even within the same subtype of lung cancer, highlighting the needs for more precise biomarkers to increase the drug efficacies.

## Discussion

In this study, we present the valuable comprehensive landscape of cancer cells, immune cells, and stromal cells in advanced NSCLC by scRNA-seq analysis. We were able to identify 11 major cell types from advanced NSCLC, including 48 subtypes besides cancer cells, the majority of which are consistent with previous studies. We focused on the cancer cells, which were not studied extensively at single-cell level in the previous literature. The shared arm-level CNAs were consistent with the observations from previous genomic sequencing data^26^, indicating a representative cohort of advanced NSCLC tumor types. Based on a quantitative approach to define inter- and intratumor heterogeneity, we unmasked a broad range of clonality, homogeneity, and the complexity beyond current classification systems of advanced NSCLC. In general, LUSC has higher ITH than LUAD. However, our data call for a more precise profiling of individual patients on the cellular levels beyond the traditional pathological definitions. For example, specimen P7 is a LUSC tumor with strong *TP63*/CK expressions and weak *NAPSA* expression (Fig. S2A). Interestingly, the major clone of this patient only represents less than 75% of its cancer cells (Fig. S3C). Further investigation showed one of its minor clones clustered together with many LUAD patients.

We also identified rare cell types such as FDC and Th17-like lymphocytes. The existence of FDC indicated the formation of lymphoid follicles, which usually correlates with favorable clinical outcomes^27^. In tumor-infiltrating CD8^+^ T cells, there are more exhausted T cells than cytotoxic T cells, which is opposite to what is observed in early stage, resectable NSCLC patients^9^. Notably, the myofibroblast to fibroblast ratio in our study was remarkably high compared to healthy or asthma lungs^28^. Thus, CAFs with myofibroblast characteristics may act as an important malignant signature for advanced stage lung cancer. Certain cell subtypes identified in this study were previously determined to be associated with drug responses. For example, *CXCL9*+ Mac was enriched in patients responding to immunotherapy^29^.

From the cellular composition analysis, we showed neutrophils to be enriched in LUSC. This phenomenon has been demonstrated by previous studies in NSCLC that neutrophils are more abundant in human LUSC compared to LUAD due to differences in TME^30^. In a subsequent study, the master transcription factor *SOX2*, a lineage-specific oncogene for squamous cell carcinomas, was found to be overexpressed and to promote tumor associated neutrophil (TANs)-accumulation by upregulating *CXCL5* (the mouse homolog of human *CXCL6*) expression^31^. Therefore, different neutrophil infiltration features were proposed to be regulated by tumor-intrinsic driver mechanisms. On the other hand, cancer and neutrophils have stronger interactions in LUAD patients. The combined observations suggested complex and diverse functions of neutrophils within TME. Our study also revealed a correlation of tumor heterogeneity and neutrophil contents. Given that separate studies showed high neutrophil contents and high tumor heterogeneity related to immunotherapy failure respectively^32–34^, our data further bridged the gap between high neutrophil contents and high tumor heterogeneity. The mechanism of interplay between tumor heterogeneity and tumor-infiltrating neutrophils might be a key element to explain the difference in immunotherapy efficacy.

By mapping cells in TME and their possible functions, by identifying more cell types and their marker genes, and by highlighting intratumor cell–cell interactions, we presented here a comprehensive collection of datasets, which provide deep insights on advanced NSCLC. Some of our findings are unreported and will need further functional validation. Despite this limitation, it can serve as valuable resources and a proof-of-concept study for future research to identify biomarkers and targets for treatment and enable personally tailored therapeutic decisions for patients with advanced NSCLC.

## Methods

### Patients

All specimens analyzed in this study were obtained from patients with histologically proven advanced, unresectable NSCLC. Smoking habits were categorized into smokers (individuals smoke >20 packs/year or <10 years of smoking cessation history prior to enrollment) and non-smokers (individuals smoke <100 cigarettes in their lifetime). All samples were collected from primary lung tumor by diagnostic procedures including transcutaneous needle biopsy or bronchoscopy from November 2018 to August 2019 and all subjects have provided their written informed consent. Histopathological review of hematoxylin-eosin stained sections was performed by senior lung pathologists. Immunohistochemistry was done for further histological subtype classification. Reverse transcription PCR (RT-PCR) was performed for testing of *EGFR* mutation*, KRAS* mutation*, ALK* fusion*, ROS1* fusion*, RET* fusion, *HER2* mutation, *BRAF* mutation, and *MET* exon14 skipping for all adenocarcinoma, some squamous carcinoma and some NSCLC patients. The study was approved by the Ethical Committee of Shanghai Pulmonary Hospital (K18-089-1).

Characteristics of patients are summarized in Fig. 1a and Supplementary Table 1. Of all 42 patients, 35 were biopsied before systemic treatment, 2 after failure of TKI, 3 after failure of immunotherapy, and 2 after failure of chemotherapy.

### Tissue dissociation and single-cell suspension preparation

Fresh samples were stored in the GEXSCOPE Tissue Preservation Solution (Singleron Biotechnologies, Nanjing, China) at 2–8 °C immediately after being collected by needle biopsy or bronchoscopy. Prior to dissociation, tissue samples were washed with Hanks Balanced Salt Solution (HBSS) for three times, minced into small pieces, and digested in 2 ml GEXSCOPE Tissue Dissociation Solution (Singleron Biotechnologies) following manufacturer’s instructions. Briefly, the specimens were digested at 37 °C for 15 min with continuous agitation. A 40-micron sterile strainer (Corning) was used to separate cells from impurities after digestion. The cells were then centrifuged at 300 × *g* 4 °C for 5 min and cell pellets were resuspended in 1 ml PBS (HyClone). Cell suspensions were counted with TC20 automated cell counter (Bio-Rad) to determine cell concentration and viability.

### Single-cell RNA-sequencing library preparation

The concentration of single-cell suspension was adjusted to 1 × 10^5^ cells/mL in PBS. Single-cell suspension was then loaded onto a microfluidic chip (GEXSCOPE Single Cell RNA-seq Kit, Singleron Biotechnologies) and scRNA-seq libraries were constructed according to the manufacturer’s instructions (Singleron Biotechnologies). The resulting scRNA-seq libraries were sequenced on an Illumina HiSeq X10 instrument with 150 bp paired end reads.

### Generation of single-cell gene expression matrices

Raw reads were processed to generate gene expression matrices by scopetools (https://anaconda.org/singleronbio/scopetools). First, read one without polyT tails were filtered, then cell barcodes and unique molecular identifiers (UMI) were extracted. Adapters and polyA tails were trimmed before aligning read two to GRCh38 with ensemble version 92 gene annotation. Second, reads with the same cell barcode, UMI and gene were grouped together to count the number of UMIs per gene per cell. Cell number was then determined based on the ‘knee’ method.

### Quality control, cell-type clustering, and major cell-type identification

We removed cells that had either lower than 200 or higher than 5000 expressed genes. Furthermore, we discarded cells with more than 30,000 UMIs and mitochondria content higher than 30%. Finally, 90,406 cells were obtained for the downstream analysis. We obtained 1673 genes and 5238 UMIs per cell on average. We opted out the batch effect correction algorithm based on the highly consistent results among patients and the undesirable removal of heterogeneity among cancer cells of individual patients (Fig. S1b). Harmony was used as the batch effect removal method.

We used Seurat 2.3 to first normalize expression matrices by function NormalizeData and ScaleData. Then FindVariable function was applied to select the top 600 variable genes and perform principle component analysis. The first 20 principle components and resolution 1.0 were used with FindClusters function to generate 37 cell clusters. To assign one of the 11 major cell types to each cluster, we scored each cluster by the normalized expressions of the following canonical markers: Endothelial cells (*CLDN5*, *VWF*, *PECAM1*), Epithelial cells (*CAPS*, *SNTN*), Alveolar cells (*CLDN18*, *AQP4*, *FLOR1*), Fibroblasts (*COL1A1*, *COL1A2*, *DCN*), T cells (*CD2*, *CD3D*, *CD3E*, *CD3G*), B cells (*CD79A*, *CD79B*), Myeloid cells (*CD14*, *LYZ*), Neutrophils (*CSF3R*, *S100A8*, *S100A9*), Follicular dendritic cells (*FDCSP*), Mast cells (*GATA2*, *TPSAB1*, *TPSB2*). The highest scored cell type was assigned to each cluster. Cancer cell clusters were negative for normal lung epithelial markers and positive for *EPCAM*. The clusters assigned to the same cell type were lumped together for the following analysis. The final results were manually examined to ensure the correctness of the results and visualized by Uniform Manifold Approximation and Projection (UMAP). The 11 major cell types were chosen by initial exploratory inspection of the differentially expressed genes (DEGs) of each cluster combined with literature study. The DEGs were generated by Seurat FindMarkers function.

### LUAD and LUSC classification based on scRNA-seq expression

We defined a LUAD and a LUSC score for each patient. The score was calculated based on the average percentage of marker expressions of tumor cells for LUAD (*NAPSA* and *TTF-1*) or LUSC (*KRT5*, *DSG*, and *TP63*), and the higher scored subtype was assigned to each patient. If both scores are less than 0.05, translating to 5% of cells expressing given markers, we assigned the patient to NSCLC. Considering both pathological subtype assignment and scRNA subtype assignment (Fig. S2A), we determined a final classification upon the review of experts. All the following grouping was based on the final classification of patients.

### scRNA-seq-based CNA detection

We inferred CNAs of 42 patients by InferCNV^14^ using single-cell transcriptomic profiles. As described in InferCNV, we used non-malignant cells including immune cells and stromal cells as baselines to estimate the CNA of malignant cells. Briefly, genes were sorted by their genomic locations on each chromosome. We then used 101 genes as a slide window to smooth the relative expression on each chromosome to remove gene-specific expression influence. Gene expressed in less than 20 cells were filtered. We centered the relative expression values to 1 and used 1.5 standard deviation of the residual normalized expression values as the ceiling and floor for visualization using R package Pheatmap. For visualization, randomly sampled 100 malignant cells of each patient were shown as their representative CNA profiles.

### Intratumoral heterogeneity scores based on CNAs and gene expressions

The calculations of intratumoral heterogeneity scores were inspired by a previous study and modified as follows^35^. First, to calculate ITH_CNA_, we used the relative expression value matrix generated by inferCNV and calculated the pairwise cell–cell distances using Pearson’s correlation coefficients for each patient. ITH_CNA_ was defined as interquartile range (IQR) of the distribution for all malignant cell pairs’ Pearson’s correlation coefficients. Similarly, we also used gene expression profiles of cancer cells of each patient to construct the distribution of the intratumoral distances. ITH_GEX_ was assigned as the IQR of the distribution. Public single-cell lung cancer datasets GSE131907 and E-MTAB-6149 were used to calculate the ITH_GEX_ scores of early-stage and advanced stage lung cancer.

### Cell subtype identification

We further clustered T cells, B cells, neutrophils, myeloid cells, fibroblasts, endothelial cells, alveolar cells, epithelial cells, and cancer cells individually. We set the resolution to 0.8 for T and B cells. For myeloid cells, endothelial cells and alveolar cells, the resolution was 0.6. For fibroblasts, neutrophils and epithelial cells, we set resolution to 0.4, 0.2, and 1.2, respectively. Within each lineage, we applied an iterative process to remove putative doublet clusters, if any, and reclustered the remaining cells. Putative doublets were identified by double positive expressions of the canonical marker genes of 11 major cell types discussed above. Within T lineage, we used the following markers for subtype identification: CD8+ exhausted T (*CD8A*, *LAG3*, and *TIGIT*), CD8+ effector T (*CD8A*, *GNLY*, *GZMA*, *GZMK*, *GZMB*, *GZMH*), CD4+ naïve T (*CCR7*, *LEF1*, *IL7R*, and *SELL*), CD4+ Tregs (*FOXP3*, *IL2RA*, and *IKZF2*), CD4+ proliferating (*TOP2A*, *MKI67*), and CD4+ Th17-like (*KLRB1*, *RORC*). Note that *CD4* itself has low RNA expression levels and CD4 T cells were deducted by CD3 positive and CD8 negative. *KLRC1*, *KLRD1*, and *NKG7* were used as the markers of NK cells. T cell subtypes were also predicted by singleR based on T cell annotations of public dataset GSE99254^9,36^. Similarly, we distinguished follicular B cells (*MS4A1*, MHC-II, *CXCR4*) from plasma cells (*MZB1*, *JCHAIN*, IgH) among the B cell lineage. Plasmacytoid DC (*IL3RA*, *LILRA4*, *CLEC4C*) was clustered in the B cell lineage. For the myeloid clusters, macrophages were positive for canonical marker *CD68*, and M2-like macrophage markers *CD163* and *MRC1*/CD206. Other myeloid cell types were confirmed by specific marker genes including classical monocytes (*CD14*, *LYZ*, *VCAN*), cDC1 (*XCR1*, *CLEC9A*), cDC2 (*FCER1A*, *CD1C*), and mature DC (*LAMP3*).

Within fibroblasts (*DCN* and *COL1A1*), *RGS5* and *CSPG4* was used to mark the pericytes. Myofibroblasts were identified by upregulation of *ACTA2* and *MYH11*. For endothelial cells (*PECAM1*, *VWF* and *ENG*), tip cells are characterized by their markers and angiogenesis-related genes (*KCNE3*, *ESM1*, *ANGPT2*, and *APLN*). Vascular cells mainly consisted of VECs and AECs, respectively identified by their markers *ACKR1* and *GJA5*. A subset of the cells expressing lymphatic markers such as *PDPN* and *PROX1* were defined as LECs-like. Using representative markers for classical airway epithelial cell types, we identified alveolar Type 2 cells (*SFTPC*, *SFTPA1*, and *ABCA3*), club cells (*SCGB1A1* and *SCGB3A1*), basal cells (*KRT5*, *KRT6A*, and *KRT14*), and ciliated cells (*FOXJ1*, *TPPP3*, and *PIFO*) for alveolar cells and other lung epithelial cells. Specific genes for cell-type identification are provided in Supplementary Table 2. For cancer clusters, clustering resolutions 0.2, 0.4, and 0.6 were all used to test the robustness of cell grouping.

### Trajectory analysis

We applied Monocle2 to determine the lineage differentiation of cell subtypes with potential developmental relationship. For CD4+ T cells, we used Seurat 2.3 FindVariableFeatures function to select top 1500 high variable genes of four CD4 clusters to order cells. DDRTree was used to learn tree-like trajectories. The heatmap along the developmental trajectory was only shown for marker genes of T subtypes. For Cancer cells and normal epithelial cells, we used AT2 cells, club cells, and LUAD cancer cell clusters to infer the evolutional paths for LUAD tumors. For LUSC tumors, we selected basal cells, club cells, and LUSC cancer cell clusters. Top 1000 high variable genes were used for both LUAD and LUSC trajectories. We also applied Slingshot to uncover the CD4+ T cell development trajectory. The identified paths were mapped to UMAP projection for visualization.

### Cellular composition analysis between patient groups

To assess whether cell-type compositions were significantly different between groups of patients, we used R package ggpubr for the statistical testing and visualization. For the comparison of two groups, *t*-test was applied to test the statistical significance. *P*-values <0.05 were considered statistically different. To assess the correlation between ITH and cellular composition, ggscatter function in ggpubr was applied to calculate the Pearson correlation coefficients and the associated p-values.

### Intercellular interaction analysis

We used CellphoneDB^37^ to perform the interaction analysis between cell types in each sample. We set the iteration to 1000 and otherwise followed the default settings of the software. The cellular network was constructed based on interactions existing in more than five patients. The interaction pairs with rank larger than 0.1 were discarded to increase the specificity. For cell interaction network, cell types were considered nodes, and the number of interactions between two cell types were treated as edge weights. The network was visualized by Cytoscape^38^. For the cell-type interaction networks, we filter the cell types with interaction lower than 500. The line width and color scale were proportional to the edge weights. Gene interaction networks were generated as following. First, a master network containing both cell types and genes was constructed. Both ligand-receptor relationship between genes and expression relationship between cell types and genes were considered edges. From the master network, cell nodes were then removed to generate the gene only networks. The first four major connected components were extracted to represent the intercellular gene interaction networks. Next, we analyzed important intercellular signals including cytokines, growth factors, and immune checkpoints. We showed the relative expression levels (z-scores) of ligands or receptors, against the percentages of patients with significant interactions for each group between cell-type pairs. We used public dataset GSE131907 to compare cellular interaction between early-stage and late-stage LUAD.

### TCGA survival analysis

We use the web server of Gene Expression Profiling Interactive Analysis (GEPIA)^39^ for TCGA survival analysis. Specifically, an interested gene name and cancer subtype were chosen as the inputs to generate the survival curves for patient overall survival (OS) and the statistical testing results. We used ‘median’ as the group cutoff metric to assign the lower and higher half of the patients as the low and high group, respectively. *P*-values <0.05 was considered statistically significant.

### Reporting summary

Further information on research design is available in the Nature Research Reporting Summary linked to this article.

## Supplementary information

Supplementary Information

Description of Additional Supplementary Files

Supplementary Data 1

Supplementary Data 2

Supplementary Data 3

Reporting Summary

## Data Availability

The raw sequencing data were deposited at Gene Expression Omnibus GSE148071. The published data used for validation or comparation in this study were retrieved from the NCBI Gene Expression Omnibus database accession code GSE131907^12^, GSE99254^9^, and ArrayExpress under Accessions E-MTAB-6149^8^. The remaining data are available within the Article, Supplementary Information or available from the authors upon request.
