# Supplementary material for: Single-cell profiling of tumor heterogeneity and the microenvironment in advanced non-small cell lung cancer
Source: Nat Commun. 2021 May 5;12:2540. doi: 10.1038/s41467-021-22801-0 (PMC8100173; doi:10.1038/s41467-021-22801-0)
Supplement: Supplementary file 1 — Supplementary Information [file 41467_2021_22801_MOESM1_ESM.pdf]

Figure S1

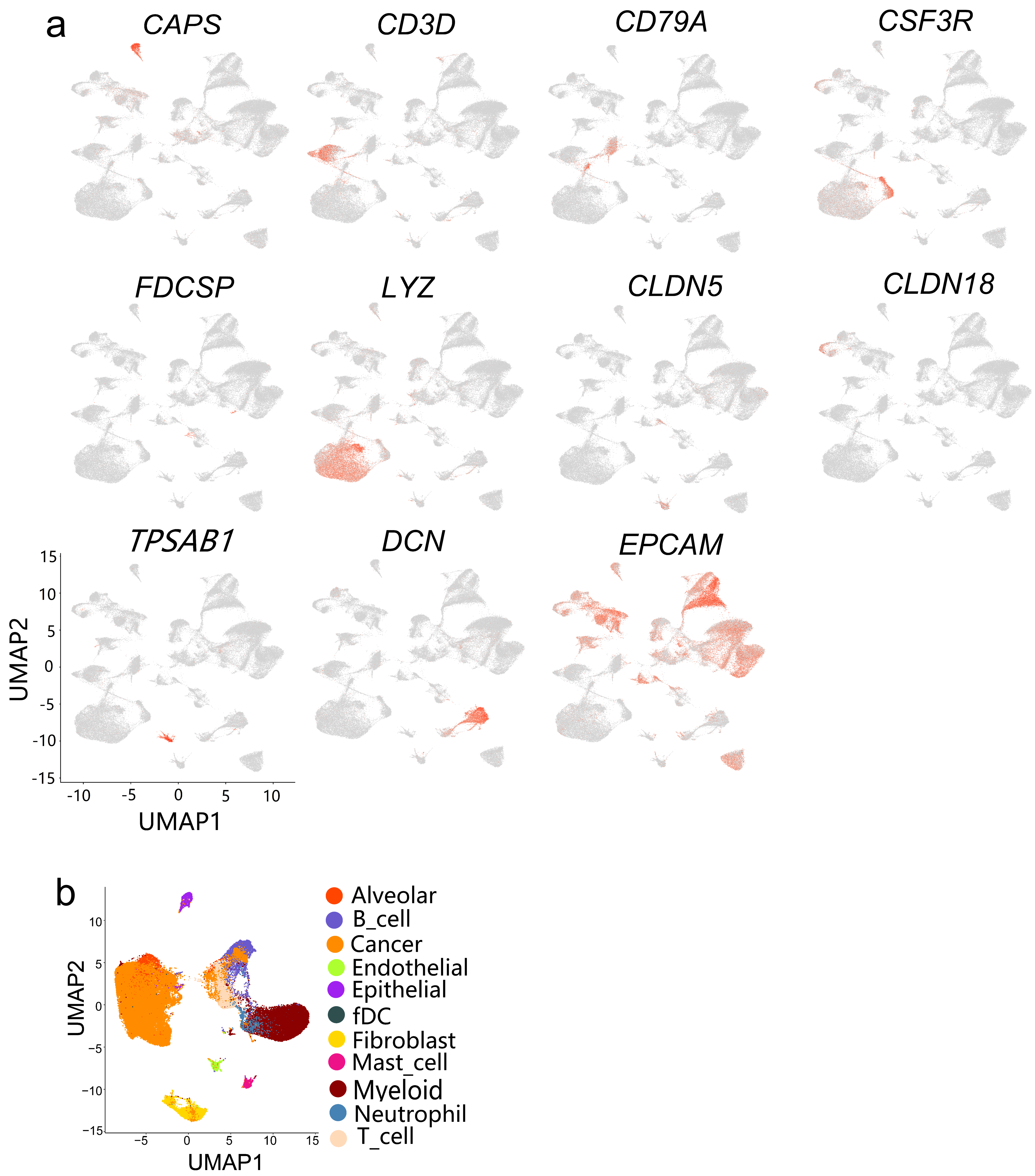

Fig. S1: 11 major cell types of 42 patients.

a) Feature plots of canonical markers of 11 major clusters, visualized by UMAP. b) UMAP of all cells after batch effect correction, colored by cell type.

Figure S2

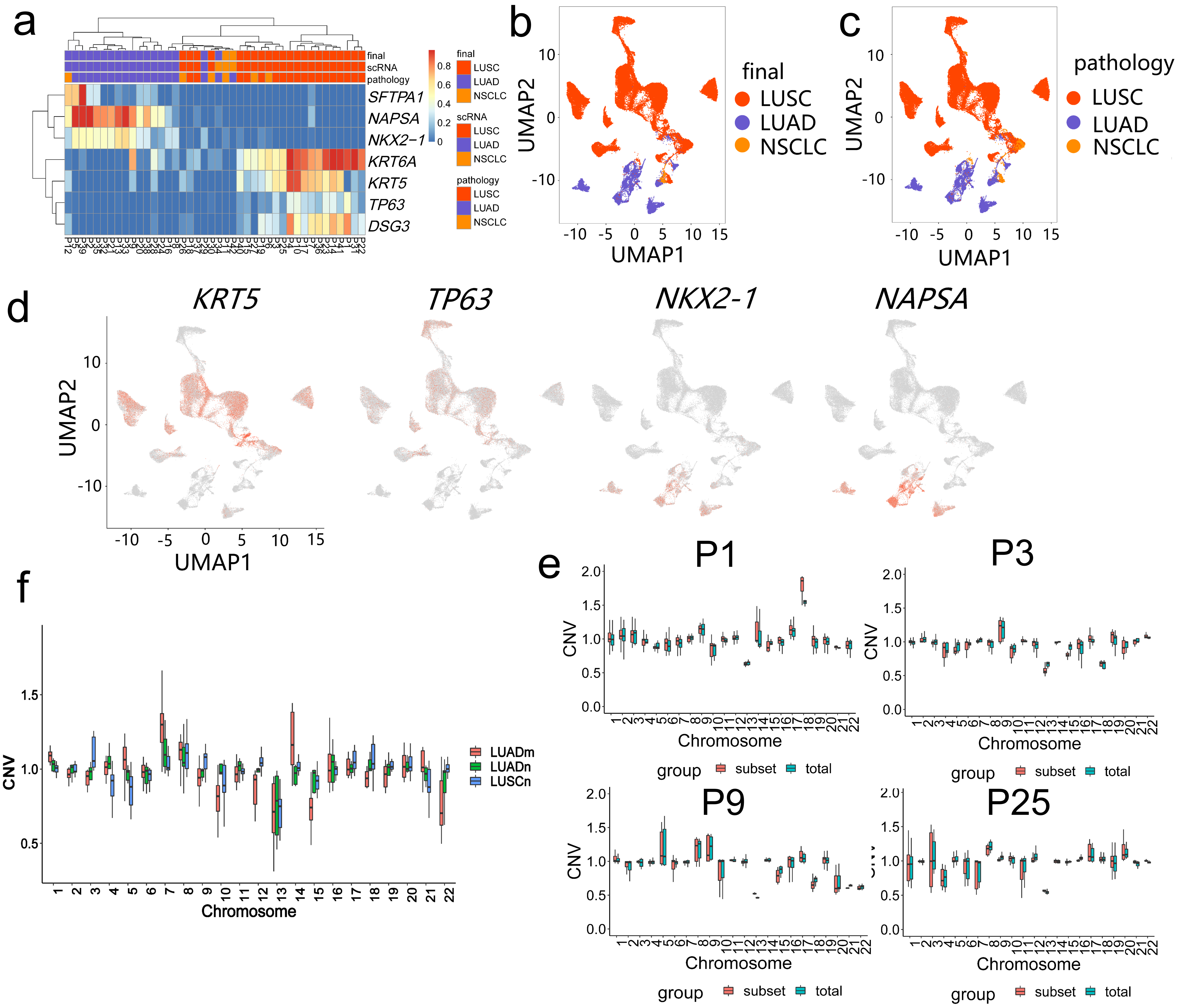

Fig. S2: Classification of patient subtypes.

a) Heatmap of LUAD and LUSC marker expression of tumor cells of all patients. The color scale was based on the percentage of expressing tumor cells for each patient. The samples were clustered by their similarities. The samples UMAP visualization of all cancer cells, colored by b) subtypes determined by pathology and c) final subtypes of each patient combining both pathology and scRNA classifications. d) Feature plots of LUAD and LUSC markers displayed on UMAP. e) Boxplots of average CNA changes of each chromosome for three groups of patients, LUADm, LUADn and LUSCn. f) The CNA profile comparison of 100 malignant cells and all malignant cells of four randomly selected patients.

In e) and f), the lower hinge, middle line and upper hinger of boxplots represented the first, second and third quartiles of the distributions. The upper and lower whiskers corresponded to the largest and smallest data points within the 1.5 interquartile range. All actual data values were also plotted as dots alongside the boxplots.

Figure S3

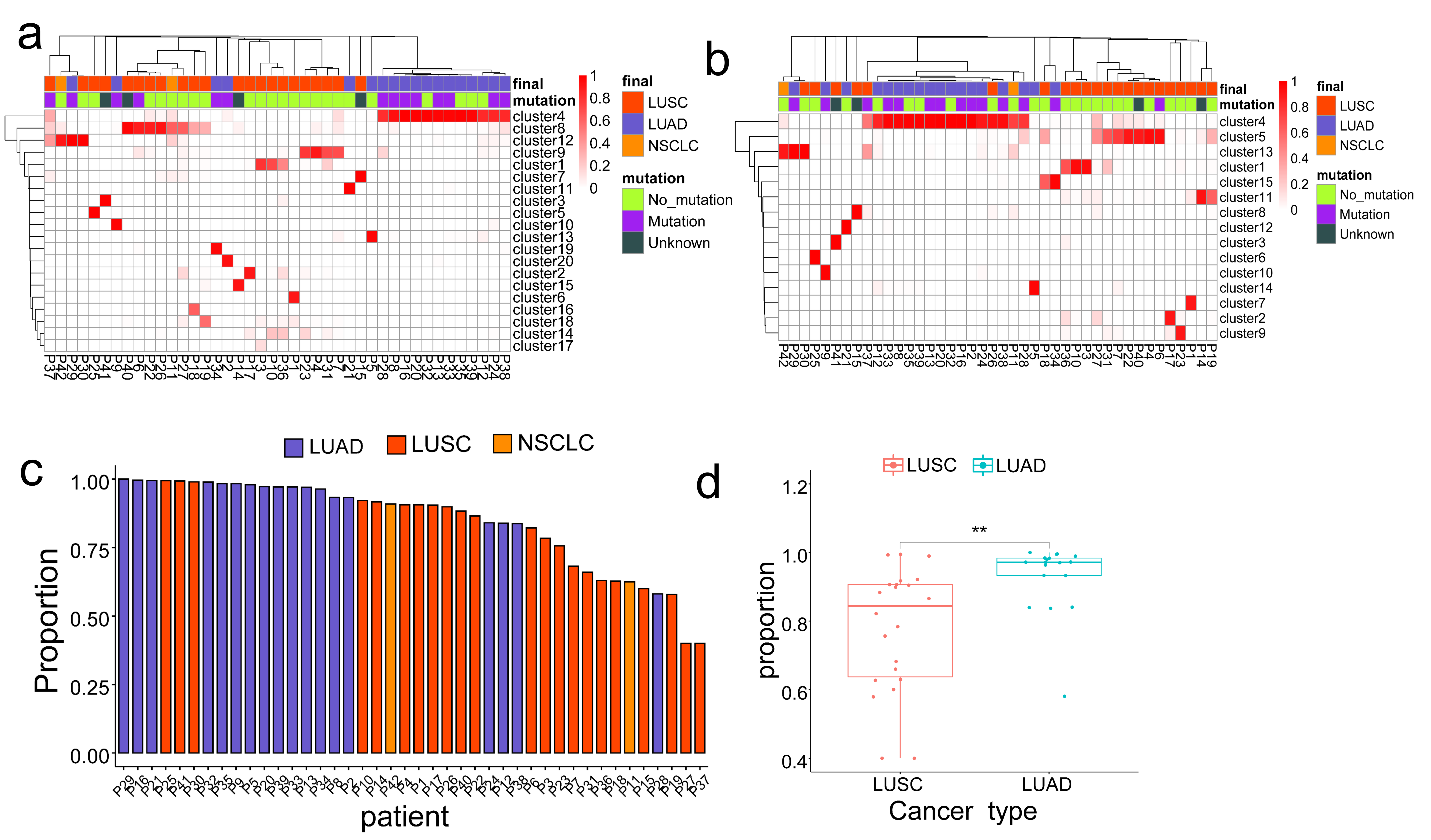

Fig. S3: Inter- and intra-tumor heterogeneity of cancer cells.

Related to Fig. 2. Heatmap displaying proportions of cancer cells of each patient in cancer clusters. The clustering results of cancer cells were generated using a) resolution 0.6 and b) 0.2 in Seurat. The arrangement of the patients on the y-axis were based on their similarities using hierarchical clustering. c) The proportion of the major clone in each patient, sorted from largest to smallest. First, the percentages of cancer cells of patients within each cluster were calculated. Then we sorted these fractions for each patient and deemed the largest number as the proportion of the major clone for each patient. When all cancer cells of a certain patient located in one cluster, the proportion of the major clone equals 1. d) The statistical test comparing the major clone proportions for LUSC and LUAD (red/LUSC: n = 22 and blue/LUAD: n = 18). Two-sided unpaired Wilcoxon test was performed to compare between groups for tests in both a and b (**p <= 0.01; *p <= 0.05; ns p > 0.05). The lower hinge, middle line and upper hinger of boxplots represented the first,

second and third quartiles of the distributions. The upper and lower whiskers corresponded to the largest and smallest data points within the 1.5 interquartile range. All actual data values were also plotted as dots alongside the boxplots.

Figure S4

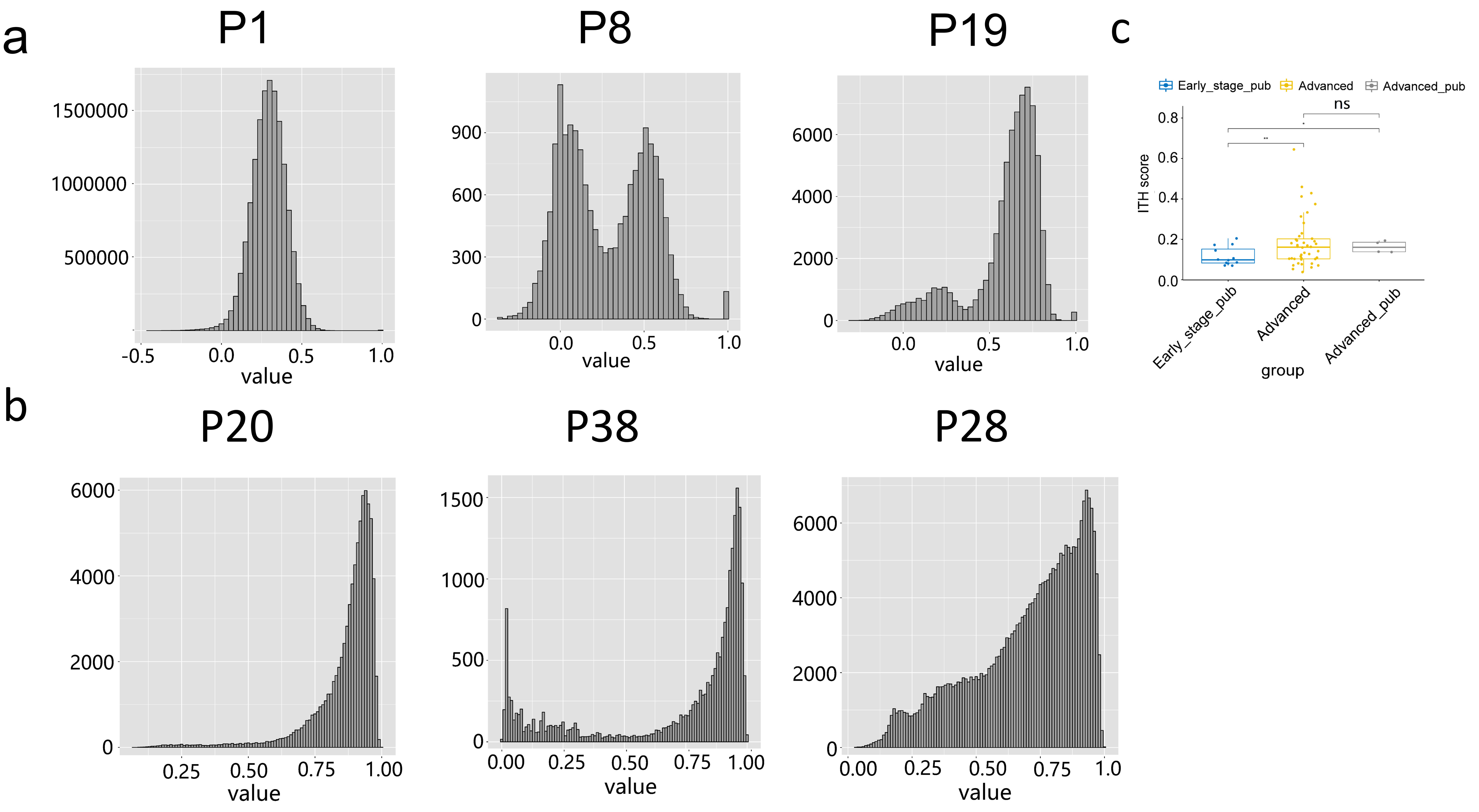

Fig. S4: Distributions of intra-tumoral distances of tumors and ITH difference between early and late stage NSCLC.

Examples of diverse shapes of intra-tumoral similarities for a) CNAs and b) expressions. Three representative patients were shown for each. c) The comparison of ITH_{GEX} between early and late stage NSCLC. Three patient populations were compared pairwise: early stage patients in public datasets (n = 12), late stage patients in public datasets (n = 4), and late stage patients in this study (n = 42). Significant levels were tested by two-sided unpaired Wilcoxon test and p values were marked as following **: p <= 0.01; *: p <= 0.05; ns: p > 0.05. The result showed increased intratumor heterogeneity of late stage patients with no apparent batch effect. The lower hinge, middle line and upper hinger of boxplots represented the first, second and third quartiles of the distributions. The upper and lower whiskers corresponded to the largest and smallest data points within the 1.5 interquartile range. All actual data values were also plotted as dots alongside the boxplots.

Figure S5

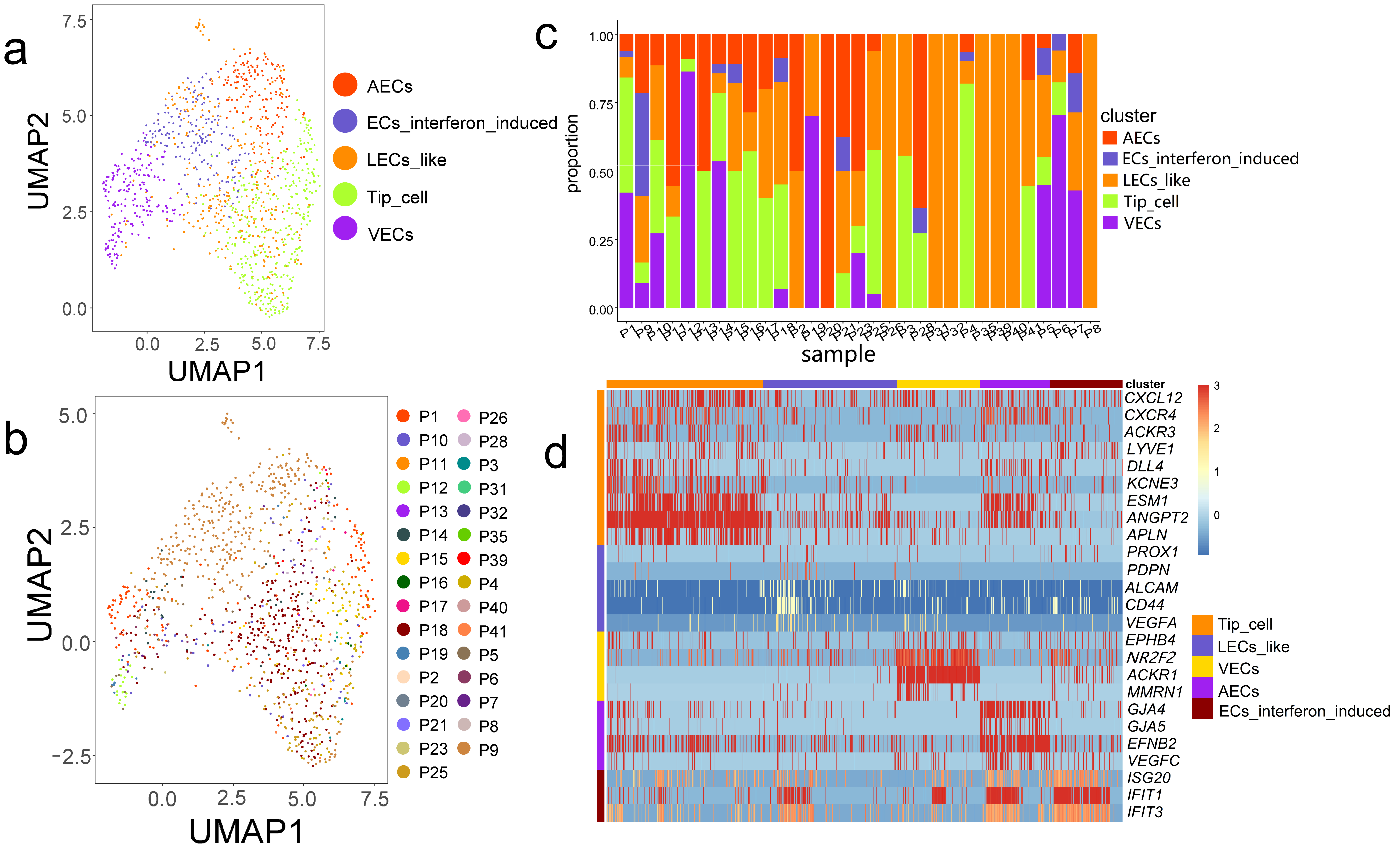

Fig. S5: Subtypes of endothelial cells.

UMAP visualization colored by a) 5 subtypes of endothelial cells and b) patient IDs. An endothelial cluster, featured by the interferon stimulated gene (ISG) signature, mainly comes from one single patient (P9) with ALK rearrangement. c) Endothelial subtype composition of each patient. Significant numbers of endothelial cells were detected in 31 out of 42 patients. Source data are provided as a Source Data file. d) Heatmap of subtype marker genes and other related genes. we identified up-regulation of chemokine CXCL12 as well as its canonical receptor CXCR7 and CXCR4 in tip cells and AECs. CXCR4 is considered as a biomarker of tumor endothelium, specifically tip cells, to increase the sprouting tumor vessels within hepatocellular carcinoma (HCC) and is associated with poor prognosis[1]. In human breast and lung cancers, CXCR7 is highly expressed in tumor-associated blood vessels but not normal vasculature[2].

Investigating the role of CXCL12-CXCR7/CXCR4 axis in cancer, it has recently been shown that specific small molecule antagonists of CXCL12, CXCR7 or CXCR4 could significantly reduce TEC angiogenesis and overall tumor burden[3]. Vascular adhesion molecules such as ALCAM and CD44, both up-regulated in the lymphatic endothelial-like cells (LECs-like), were reported to support tumor growth, metastasis and endothelial-to-mesenchymal transition (EMT) [4-6].

Figure S6

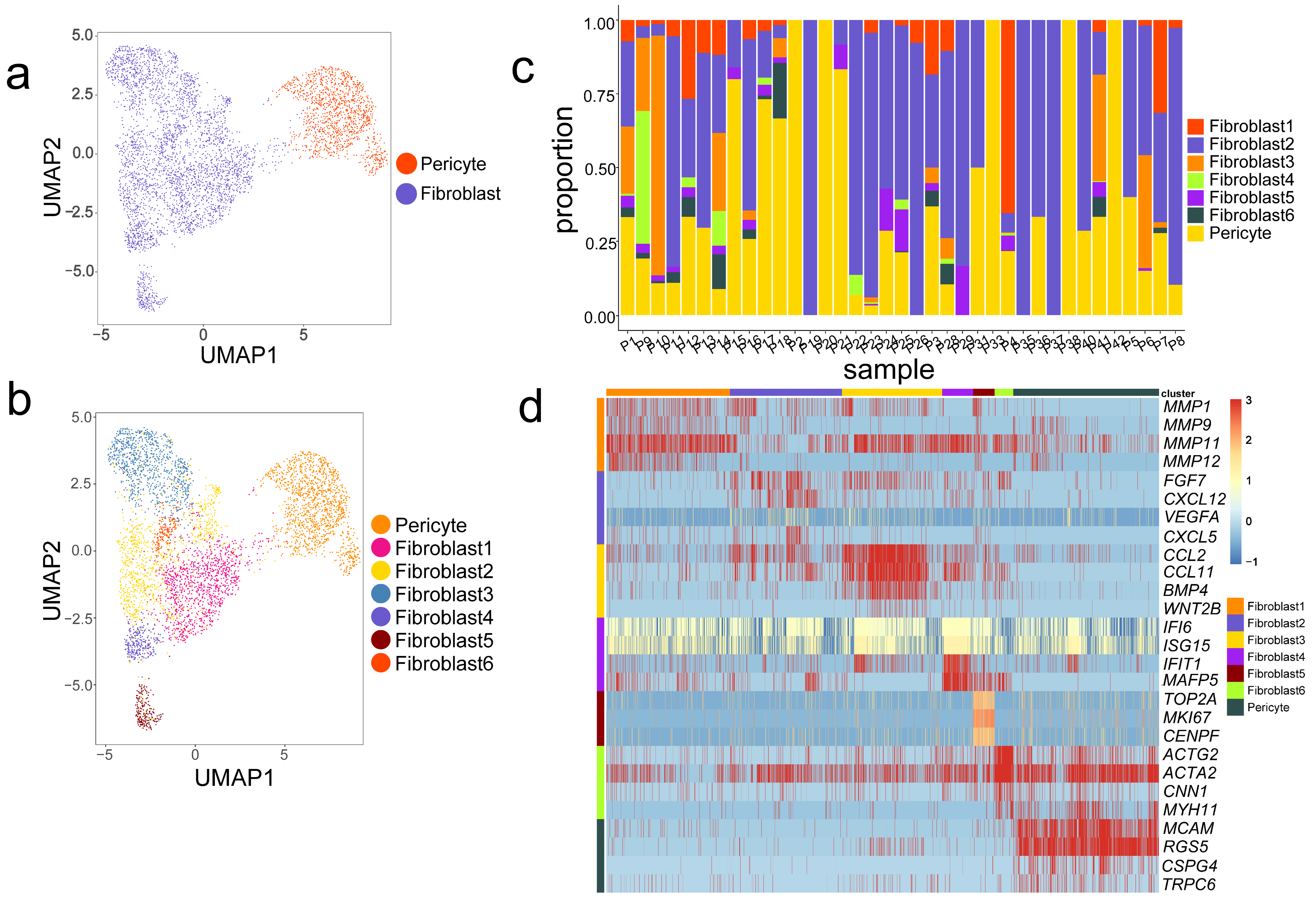

Fig. S6: Subtypes of fibroblasts.

UMAP visualizations of a) fibroblasts and pericytes, and b) 6 fibroblast subtypes. Fibroblasts were divided to pericytes, fibroblasts, and myofibroblasts. Fibroblasts and myofibroblasts were similar with respect to production of extracellular matrix, and to smooth muscle related genes. c) Fibroblast subtypes and pericytes composition of each patient. Fibroblasts (3,673 cells) and pericytes (1,315 cells) were detected in 37 out of 42 patients. Source data are provided as a Source Data file. d) Heatmap shown marker genes and other important gene expressions in pericytes and fibroblast subtypes. Notably, cluster 2 and 5 are remarkably patient-specific and proposed to be CAFs marked by the expression of pro-tumor molecules, such as matrix metallopeptidases (MMPs), chemokines and MFAP5. MMPs upregulated in cluster 2, such as MMP-1, MMP-9, MMP-11, MMP-12, are efficient extracellular matrix molecules showing pro-tumorigenic function in various tumor types[7]. Fibroblast cluster 5 from patient P9 revealed high

expression levels of several ISGs including IFI6, ISG15, and IFIT1, in line with the expression signature of the endothelial cells from the same patient. Cluster 4 revealed high expression of inflammatory chemokines such as CCL2, CCL11, CXCL14 and may contribute to the recruitment of tumor-associated macrophages into the tumor microenvironment[7,8]. For CAFs, CXCL14 and CCL2 were also expressed by fibroblasts in healthy lung and distal non-malignant lung samples' respectively[9]. These results suggested that CAFs execute complicated and diverse pro-tumorigenic programs. The ISG signature has been proposed to be tumor-related and contribute to the resistance of cancer therapy[10].

Figure S7

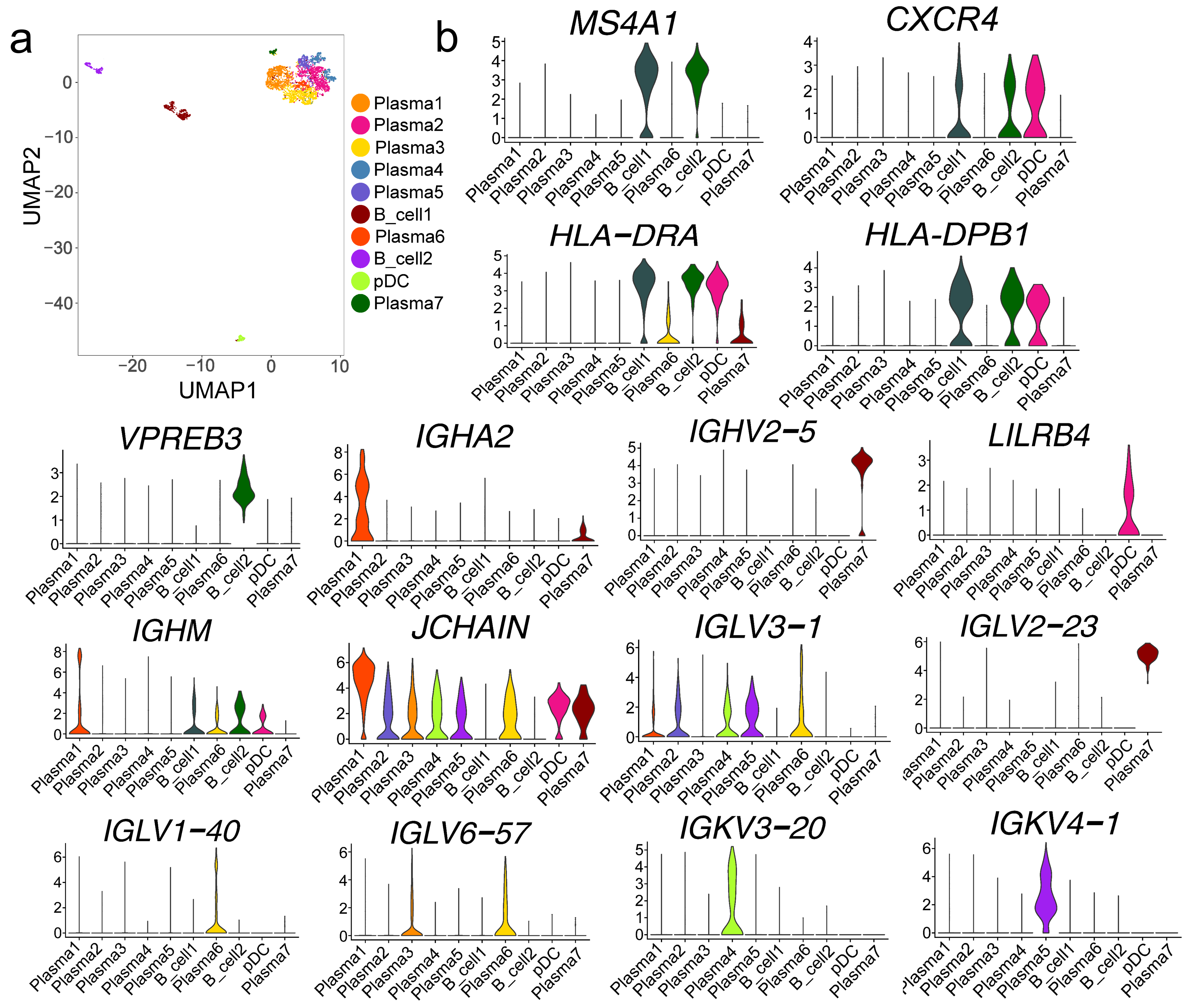

Fig. S7: Subtypes of B cells.

a) UMAP visualization of B cells, plasma cells and pDCs. We detected 2 clusters of B cells and 7 clusters of plasma cells and a pDC cluster. b) Violin plots of important genes of B and plasma cells. Plasma cells predominantly belong to the IgG class. Within IgG plasma cells, we observed enrichment of various B cell receptor V regions, such as heavy chain genes (IGHV2-5 and IGHV3-13), light chain lambda genes (IGLV6-57, IGLV3-1, IGLV1-40, IGLV1-44 and IGLV2-23), and light chain kappa genes (IGKV4-1). Plasma1 displayed high expression of JCHAIN, IGHM and IGHA1/2. Different from early-stage lung cancer, plasma cells are more abundant than follicular B cells[11]. Two follicular B cell

subpopulations showed significant expression of the pre-B cell marker VPREB3 which is normally expressed by precursor B cells in bone marrow and by a subset of normal germinal center B cells in secondary lymphoid organs[12].

Figure S8

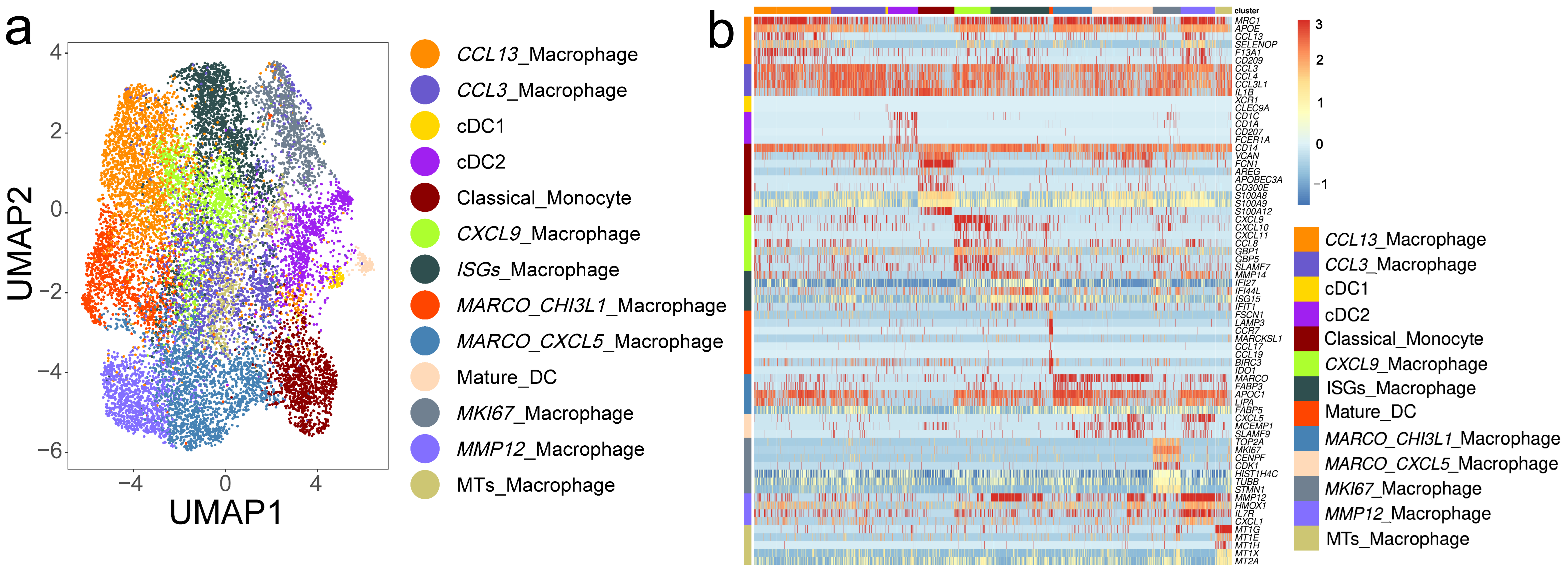

Fig. S8: Subtypes of myeloid cells.

a) UMAP plot displaying subtypes of myeloid lineage. b) Heatmap of marker genes for each myeloid subtype. We observed subsets of monocytes, macrophages, and DCs. Macrophages comprised the most abundant myeloid subset with 9 distinct clusters based on our analysis. Notably, all these nine different macrophages subpopulations showed pro-tumor M2-like (MRC1, APOE) and M1-like (HLA) signatures simultaneously. We renamed these clusters by their specifically up-regulated genes. MARCO was uniquely expressed in two populations. These macrophages are suggested to be tissue resident alveolar macrophages[13]. Other macrophage populations displayed different expression levels for diverse chemokines. CXCL9_macrophages had CXCL9, CXCL10 and CXCL11 upregulated. All three chemokines have a common receptor CXCR3, which is highly expressed on activated T cells. Such macrophages are implicated in recruiting effector T cell into tumors[14,15]. CCL3_macrophages showed upregulation of proinflammatory chemokines such as CCL3, CCL4 and CCL5 and were associated with an M1 phenotype, suggesting potential inhibition of tumor progression[16-18]. MKI67_macrophages were characterized as proliferating cells by the cell cycle-related markers of TOP2A, MKI67 and CDK1.

Figure S9

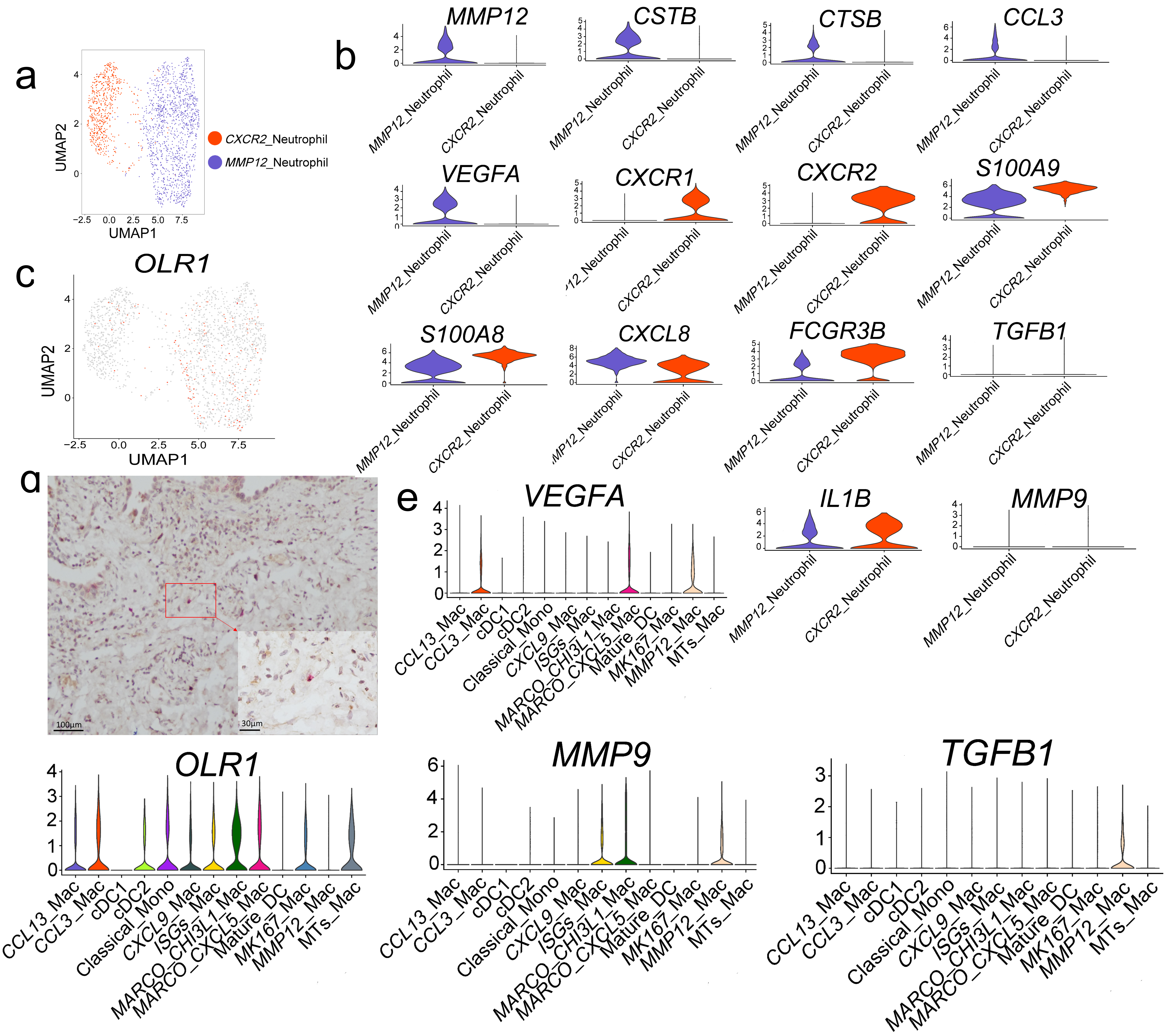

Fig. S9: Subtypes of neutrophils.

a) Two neutrophil subtypes visualized by UMAP projection. b) Violin plots of marker genes and other important genes of two neutrophil subtypes. c) Feature plot of LOX-1 (OLR1) expression in two neutrophil clusters. d) Representative immunohistochemical (IHC) staining of CD15 (red) and LOX-1 (brown). CD15 is a canonical protein marker for neutrophils, and the results confirmed co-expression of CD15 and LOX-1. The experiments were performed for three patients, and three replicates were done for each patient. e) The violin plots of selected MDSC markers in myeloid subclusters. (Macrophages: Mac, Monocytes: Mono)

Neutrophils were clustered into 2 subpopulations with expression of canonical neutrophil markers as well as CSTB, CTSB, and IRAK2, which were tumor specific in mice NSCLC[19]. Genes including CXCR2, MMP12, VEGFA and IL1B also revealed enhanced expression. Previous studies have identified CXCR2+ PMN-MDSC migration into TME through interacting with tumor-secreted ligands in colorectal and bladder cancers[20-22]. The proinflammatory cytokines and endothelial growth factors produced in lung cancer are known to induce tumor progression and angiogenesis[23]. IL1B have been identified as a PMN-MDSC marker in a mouse mammary cancer model[24]. Both subpopulations expressed lectin-type oxidized LDL receptor 1 (LOX-1/ OLR1), which was a potential marker to distinguish PMN-MDSC from normal neutrophils[25-27]. Co-expression of CD15 (red) and LOX-1 (brown) identified by IHC with 3 repetitions supported the expression of reported PMN-MDSC marker LOX-1 in the neutrophil population on the protein level. Several widely recognized MDSC markers like VEGFA, TGFB1, MMP9 and also LOX-1 were selectively expressed by some of the myeloid subpopulations including OLR1[28-31]. However, the specificity of these reported MDSC markers in PMN-MDSCs and M-MDSCs is still unclear. Given that MDSC is a highly heterogeneous population and the scRNA-seq study of MDSC is still lacking, its characterization in cancers by transcriptomics is still a challenge. Therefore, the two neutrophil clusters were transcriptionally different with differentially expressed PMN-MDSC related genes.

Figure S10

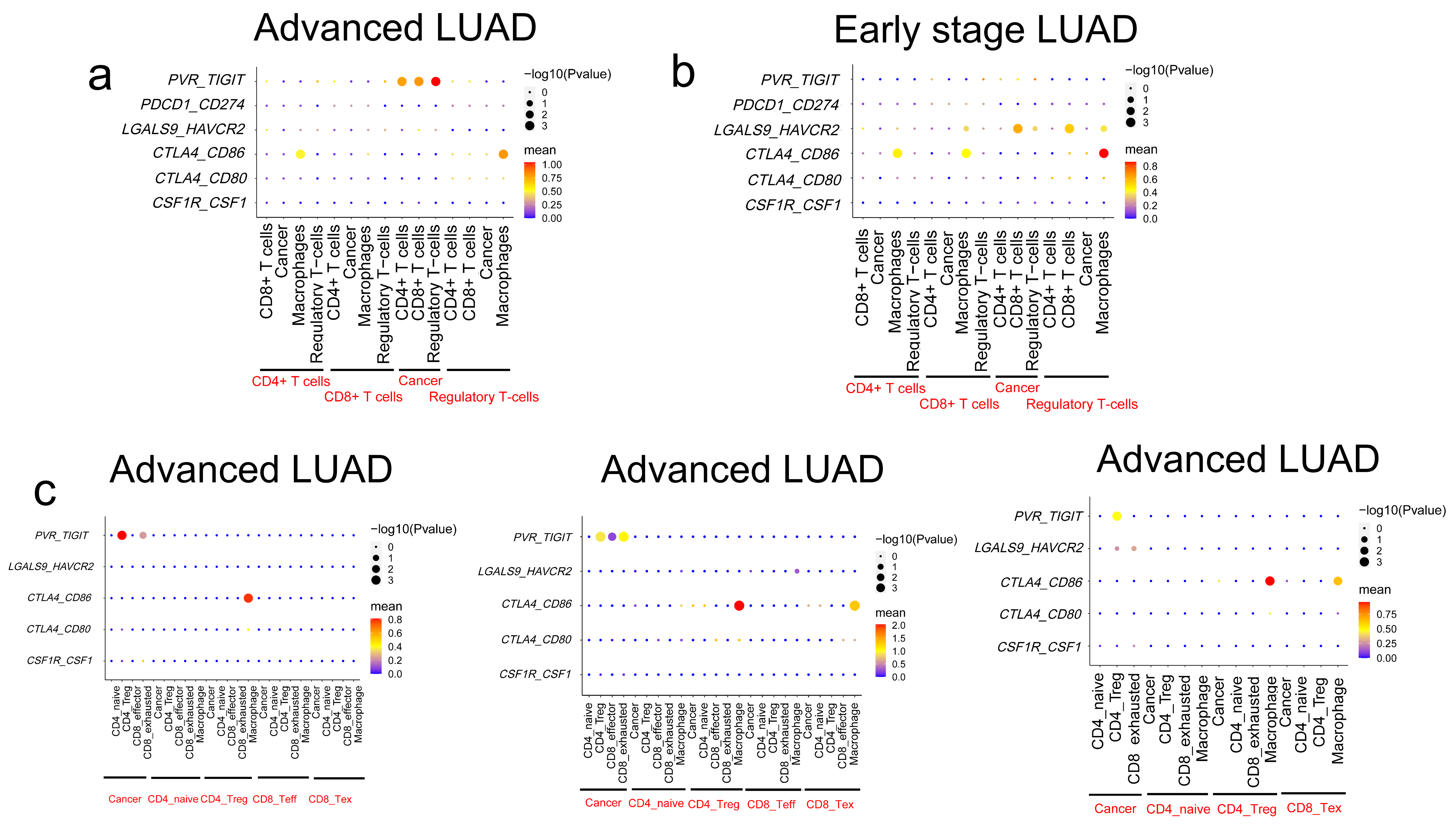

Fig. S10: Selected cellular interactions of checkpoint inhibitors in a public dataset.

Mean expression levels were represented by color (red to blue), and dot sizes displayed the negative log10 transformed p value of each ligand-receptor pair. a) An advanced LUAD patient from public dataset b) An early-staged LUAD patient from public dataset c) Advanced LUAD patients from our dataset. In all three panels, p values were obtained through 1000 times random permutation of cell type labels by cellphoneDB.

SUPPLEMENTARY TABLES

Supplementary table 1. Baseline characteristics of 42 advanced NSCLC patients.

Baseline Characteristics	N (%) (n=42)
Mean age	57.8 (35-77)
Gender	
Male	33 (78.6)
Female	9 (21.4)
Performance status	
0	4 (9.5)
1	38 (90.5)
Smoker	21 (50)
Former smoker	3 (7.1)
Non-smoker	18 (42.9)
Histology subtype	
Adenocarcinoma	18 (42.9)
Squamous carcinoma	18 (42.9)
Non-small cell lung cancer	6 (14.3)
Stage	
III b/c	14 (33.3)
IV	28 (66.7)
Driver genes	
EGFR	9 (21.4)
ALK	2 (4.8)
RET	1 (2.4)
HER2	2 (4.8)
Non-driver	24 (57.1)
Unknown	4 (9.5)
Time of biopsy	
Before systematic treatment	35 (83.3)
After failure to TKI	2 (4.8)
After failure to immunotherapy	3 (7.1)
After failure to chemotherapy	2 (4.8)

Supplementary table 2. Canonical marker genes used for cell type identification and relevant literature.

Compartment	marker gene	Cell type	PMID
Immune	*CD2*	T cells	30388455, 31501550
Immune	*CD3D/E/G*	T cells	28854175, 31501550
Immune	*KLRC1*	Natural killer cells	29610856, 31359002
Immune	*KLRD1*	Natural killer cells	31477722, 32066974
Immune	*NKG7*	Natural killer cells, CD8+ effector T cells	28091601, 29942094, 31915375
Immune	*CD8A*	CD8+ T cells	30568305, 30388455
Immune	*CD4*	CD4+ T cells	30568305, 30388455
Immune	*GNLY*	CD8+ effector T	31892341, 29942094
Immune	*GZMA*	CD8+ effector T	31915375, 29942094,30568305
Immune	*GZMB*	CD8+ effector T	31892341, 29942094
Immune	*GZMK*	CD8+ effector T	31915375, 30093720
Immune	*GZMH*	CD8+ effector T	31915375, 29942094
Immune	*CCR7*	Naïve T cells	31624246, 30568305, 31802004
Immune	*LEF1*	Naïve T cells	31624246, 29942094
Immune	*IL7R*	Naïve T cells	30568305, 31802004
Immune	*SELL*	Naïve T cells	31624246, 30568305, 29942094, 31802004
Immune	*LAG3*	Exhausted T cells	31624246, 29942094, 29942094

Immune	*TIGIT*	Exhausted T cells	29961579, 29942094, 31359002, 31802004
Immune	*FOXP3*	Regulator T cells	29434354, 30568305,31209404
Immune	*IL2RA*	Regulator T cells	29434354, 30568305
Immune	*IKZF2*	Regulator T cells	29942094, 31209404
Immune	*CTLA4*	Exhausted T cells, Regulator T cells	29434354, 31624246
Immune	*ITGAE*	Tissue-resident memory T cells	31209404, 29942094
Immune	*ITGA1*	Tissue-resident memory T cells	31209404, 29942094
Immune	*ZNF683*	Tissue-resident memory T cells	31209404, 29942094
Immune	*CD79A/B*	B cells	31604687, 30388455
Immune	*MS4A1*	Follicular B cells	29988129, 30104629
Immune	*HLA-DRs*	Follicular B cells	29988129, 30104629
Immune	*CXCR4*	Follicular B cells	29988129
Immune	*MZB1*	Plasma cells	31221805, 31209404
Immune	*JCHAIN*	Plasma cells	31892341, 31915375
Immune	*IGHG1*	Plasma cells	29988129, 31892341
Immune	*LYZ*	Myeloid cells	29988129, 31937773, 31501550
Immune	*CSF3R*	Neutrophils	31754025, 30979687
Immune	*S100A8/9*	Neutrophils	31892341, 31915375, 30967541
Immune	*FCGR3B*	Neutrophils	31754025, 31604275
Immune	*XCR1*	Conventional type 1 dendritic cell (cDC1)	32302573, 31892341

Immune	*CLEC9A*	Conventional type 1 dendritic cell (cDC1)	32302573, 31892341
Immune	*FCER1A*	Conventional type 2 dendritic cell (cDC2)	32302573, 31892341
Immune	*CD1C*	Conventional type 2 dendritic cell (cDC2)	32302573, 31892341
Immune	*LAMP3*	Mature dendritic cells	15814636, 31345789
Immune	*FDCSP*	Follicular dendritic cells	12193705, 17548624
Immune	*CD68*	Macrophages	23293084, 29017955
Immune	*MRC1*	M2 macrophages/Alternatively activated macrophages	31108906, 29017955
Immune	*CD163*	M2 macrophages/Alternatively activated macrophages	31209404, 31108906, 32302573
Immune	*CD14*	Monocytes	32302573, 31892341
Immune	*FCN1*	Monocytes	32302573, 31892341
Immune	*TPSAB1*	Mast cells	31221805, 32302573
Immune	*TPSB2*	Mast cells	31221805, 32302573
Immune	*GATA2*	Mast cells	32302573, 32385277, 25855601
Immune	*IL3RA*	Plasmacytoid dendritic cells	32302573, 31892341
Immune	*LILRA4*	Plasmacytoid dendritic cells	29313948, 32302573
Immune	*CLEC4C*	Plasmacytoid dendritic cells	29313948, 31892341
Vessel	*CLDN5*	Endothelial cells	31221805, 32385277
Vessel	*PECAM1*	Endothelial cells	31935371, 31892341
Vessel	*VWF*	Endothelial cells	31935371, 31892341
Vessel	*DLL4*	Tip cells	32060101, 20651738
Vessel	*KCNE3*	Tip cells	31754927

Vessel	*ESM1*	Tip cells	29986945, 24025447
Vessel	*ANGPT2*	Tip cells	20651738
Vessel	*ACKR1*	Vein endothelial cells	31935371, 19060902, 29986945
Vessel	*GJA5*	Artery endothelial cells	31935371, 29986945
Vessel	*PROX1*	Lymphatic endothelial cells	29988129, 31935371, 29986945
Vessel	*PDPN*	Lymphatic endothelial cells	29988129, 31935371, 29986945
Vessel	*RGS5*	Pericytes	29988129, 31221805
Vessel	*CSPG4*	Pericytes	30213051, 28536635
Stroma	*DCN*	Fibroblasts	29988129, 31197017
Stroma	*COL1A1/2*	Fibroblasts	31604275, 31221805, 29420258
Stroma	*ACTA2*	Myofibroblasts	29590628, 31462402, 31299246
Stroma	*MYH11*	Myofibroblasts	29590628, 30315278
Epithelium	*CAPS*	Epithelial cells	29988129, 28238698
Epithelium	*SNTN*	Epithelial cells	29988129
Epithelium	*CLDN18*	Alveolar cells	29988129, 29400691
Epithelium	*AQP4*	Alveolar cells	29988129, 10619865
Epithelium	*CAV1*	Alveolar type 1 cells	29988129, 26985677
Epithelium	*AGER*	Alveolar type 1 cells	29988129, 30770807,31209336

Epithelium	*SFTPC*	Alveolar type 2 cells	30760489, 29420258, 31209336
Epithelium	*SFTPA1*	Alveolar type 2 cells	27588449, 31866069
Epithelium	*ABCA3*	Alveolar type 2 cells	27942595, 29988129
Epithelium	*SCGB1A1*	Club cells	29988129, 31892341, 30967541
Epithelium	*SCGB3A1*	Club cells	30967541, 31892341
Epithelium	*KRT5*	Basal cells	30554520, 31892341,31209336
Epithelium	*KRT6A*	Basal cells	27423691, 28246210
Epithelium	*KRT14*	Basal cells	27423691, 28246210,24787069
Epithelium	*FOXJ1*	Ciliated cells	31844660, 31209336
Epithelium	*TPPP3*	Ciliated cells	30554520, 31221805
Epithelium	*PIFO*	Ciliated cells	30224385, 31209336

Supplementary References

1. Salazar, N. & Zabel, B.A. Support of Tumor Endothelial Cells by Chemokine Receptors. *Frontiers in immunology* **10**, 147 (2019).
2. Miao, Z.*, et al.* CXCR7 (RDC1) promotes breast and lung tumor growth in vivo and is expressed on tumor-associated vasculature. *Proceedings of the National Academy of Sciences of the United States of America* **104**, 15735-15740 (2007).
3. Yamada, K.*, et al.* CXCL12-CXCR7 axis is important for tumor endothelial cell angiogenic property. *International journal of cancer* **137**, 2825-2836 (2015).
4. Willrodt, A.H.*, et al.* Stromal Expression of Activated Leukocyte Cell Adhesion Molecule Promotes Lung Tumor Growth and Metastasis. *The American journal of pathology* **187**, 2558-2569 (2017).
5. Iolyeva, M.*, et al.* Novel role for ALCAM in lymphatic network formation and function. *FASEB journal : official publication of the Federation of American Societies for Experimental Biology* **27**, 978-990 (2013).
6. Agrawal, V. & Hemnes, A.R. CD44 and xCT: The Silver Bullet for Endothelial-to-Mesenchymal Transition in Pulmonary Arterial Hypertension? *American journal of respiratory cell and molecular biology* **61**, 281-283 (2019).
7. Hegab, A.E.*, et al.* Effect of FGF/FGFR pathway blocking on lung adenocarcinoma and its cancer-associated fibroblasts. *The Journal of pathology* **249**, 193-205 (2019).
8. Sjoberg, E.*, et al.* A Novel ACKR2-Dependent Role of Fibroblast-Derived CXCL14 in Epithelial-to-Mesenchymal Transition and Metastasis of Breast Cancer. *Clinical cancer research : an official journal of the American Association for Cancer Research* **25**, 3702-3717 (2019).
9. Vieira Braga, F.A.*, et al.* A cellular census of human lungs identifies novel cell states in health and in asthma. *Nature medicine* **25**, 1153-1163 (2019).
10. Benci, J.L.*, et al.* Opposing Functions of Interferon Coordinate Adaptive and Innate Immune Responses to Cancer Immune Checkpoint Blockade. *Cell* **178**, 933-948 e914 (2019).
11. Lambrechts, D.*, et al.* Phenotype molding of stromal cells in the lung tumor microenvironment. *Nature medicine* **24**, 1277-1289 (2018).
12. Rodig, S.J., et al. The pre-B-cell receptor associated protein VpreB3 is a useful diagnostic marker for identifying c-MYC translocated lymphomas. Haematologica 95, 2056-2062 (2010).
13. Aran, D.*, et al.* Reference-based analysis of lung single-cell sequencing reveals a transitional profibrotic macrophage. *Nature immunology* **20**, 163-172 (2019).
14. Chow, M.T.*, et al.* Intratumoral Activity of the CXCR3 Chemokine System Is Required for the Efficacy of Anti-PD-1 Therapy. *Immunity* **50**, 1498-1512 e1495 (2019).
15. Ding, Q.*, et al.* CXCL9: evidence and contradictions for its role in tumor progression. *Cancer medicine* **5**, 3246-3259 (2016).
16. Zeiner, P.S.*, et al.* Distribution and prognostic impact of microglia/macrophage subpopulations in gliomas. *Brain pathology* **29**, 513-529 (2019).

17. Cohen, M., *et al.* Lung Single-Cell Signaling Interaction Map Reveals Basophil Role in Macrophage Imprinting. *Cell* **175**, 1031-1044 e1018 (2018).
18. Pellizzari, G., *et al.* IgE re-programs alternatively-activated human macrophages towards pro-inflammatory anti-tumoural states. *EBioMedicine* **43**, 67-81 (2019).
19. Zilionis, R., *et al.* Single-Cell Transcriptomics of Human and Mouse Lung Cancers Reveals Conserved Myeloid Populations across Individuals and Species. *Immunity* **50**, 1317-1334 e1310 (2019).
20. Katoh, H., *et al.* CXCR2-expressing myeloid-derived suppressor cells are essential to promote colitis-associated tumorigenesis. *Cancer cell* **24**, 631-644 (2013).
21 Liao, W., *et al.* KRAS-IRF2 Axis Drives Immune Suppression and Immune Therapy Resistance in Colorectal Cancer. *Cancer cell* **35**, 559-572 e557 (2019).
22. Zhang, H., *et al.* CXCL2/MIF-CXCR2 signaling promotes the recruitment of myeloid-derived suppressor cells and is correlated with prognosis in bladder cancer. *Oncogene* **36**, 2095-2104 (2017).
23. Altorki, N.K., *et al.* The lung microenvironment: an important regulator of tumour growth and metastasis. *Nature reviews. Cancer* **19**, 9-31 (2019).
24. Alshetaiwi, H., et al. Defining the emergence of myeloid-derived suppressor cells in breast cancer using single-cell transcriptomics. Science immunology 5(2020).
25. Zhou, J., Nefedova, Y., Lei, A. & Gabrilovich, D. Neutrophils and PMN-MDSC: Their biological role and interaction with stromal cells. *Seminars in immunology* **35**, 19-28 (2018).
26. Tcyganov, E., Mastio, J., Chen, E. & Gabrilovich, D.I. Plasticity of myeloid-derived suppressor cells in cancer. *Current opinion in immunology* **51**, 76-82 (2018).
27. Veglia, F., Perego, M. & Gabrilovich, D. Myeloid-derived suppressor cells coming of age. *Nature immunology* **19**, 108-119 (2018).
28. Vetsika, E.K., Koukos, A. & Kotsakis, A. Myeloid-Derived Suppressor Cells: Major Figures that Shape the Immunosuppressive and Angiogenic Network in Cancer. *Cells* **8**(2019).
29. Gabrilovich, D.I. Myeloid-Derived Suppressor Cells. *Cancer immunology research* **5**, 3-8 (2017).
30. Bronte, V., *et al.* Recommendations for myeloid-derived suppressor cell nomenclature and characterization standards. *Nat Commun* **7**, 12150 (2016).
31. Toh, B., *et al.* Mesenchymal transition and dissemination of cancer cells is driven by myeloid-derived suppressor cells infiltrating the primary tumor. *PLoS biology* **9**, e1001162 (2011).
