# Supplementary material for: Single-cell profiling of tumor heterogeneity and the microenvironment in advanced non-small cell lung cancer
Source: Nat Commun. 2021 May 5;12:2540. doi: 10.1038/s41467-021-22801-0 (PMC8100173; doi:10.1038/s41467-021-22801-0)
Supplement: Supplementary file 2 — Description of Additional Supplementary Files [file 41467_2021_22801_MOESM2_ESM.pdf]

Description of Additional Supplementary Files

File Name: Supplementary Data 1
Description: Cell numbers of each patient for 11 major cell types in Fig. 1e. The cell numbers and portions of cancer, stromal and immune cells varied greatly among the 42 samples.

File Name: Supplementary Data 2
Description: Cell numbers of each patient for endothelial subtypes in Fig. S5c. A total of 1,183 endothelial cells were detected in 31 out of 42 patients.

File Name: Supplementary Data 3
Description: Cell numbers of each patient for Fibroblast subtypes in Fig. S6c. Fibroblasts (3,673 cells) and pericytes (1,315 cells) were detected in 37 out of 42 patients
