# Supplementary material for: Single-cell profiling of tumor heterogeneity and the microenvironment in advanced non-small cell lung cancer
Source: Nat Commun. 2021 May 5;12:2540. doi: 10.1038/s41467-021-22801-0 (PMC8100173; doi:10.1038/s41467-021-22801-0)
Supplement: Supplementary file 6 — Reporting Summary [file 41467_2021_22801_MOESM6_ESM.pdf]

nature research

Corresponding author(s): Caicun Zhou

Last updated by author(s): Jan 24, 2021

Reporting Summary

Nature Research wishes to improve the reproducibility of the work that we publish. This form provides structure for consistency and transparency in reporting. For further information on Nature Research policies, see Authors & Referees and the Editorial Policy Checklist .

Statistics

For all statistical analyses, confirm that the following items are present in the figure legend, table legend, main text, or Methods section.

n/a	Confirmed	
☐	☒	The exact sample size (*n*) for each experimental group/condition, given as a discrete number and unit of measurement
☐	☒	A statement on whether measurements were taken from distinct samples or whether the same sample was measured repeatedly
☐	☒	The statistical test(s) used AND whether they are one- or two-sided<br>*Only common tests should be described solely by name; describe more complex techniques in the Methods section.*
☒	☐	A description of all covariates tested
☐	☒	A description of any assumptions or corrections, such as tests of normality and adjustment for multiple comparisons
☐	☒	A full description of the statistical parameters including central tendency (e.g. means) or other basic estimates (e.g. regression coefficient) AND variation (e.g. standard deviation) or associated estimates of uncertainty (e.g. confidence intervals)
☐	☒	For null hypothesis testing, the test statistic (e.g. *F*, *t*, *r*) with confidence intervals, effect sizes, degrees of freedom and *P* value noted<br>*Give P values as exact values whenever suitable.*
☒	☐	For Bayesian analysis, information on the choice of priors and Markov chain Monte Carlo settings
☒	☐	For hierarchical and complex designs, identification of the appropriate level for tests and full reporting of outcomes
☐	☒	Estimates of effect sizes (e.g. Cohen's *d*, Pearson's *r*), indicating how they were calculated

Our web collection on statistics for biologists contains articles on many of the points above.

Software and code

Policy information about availability of computer code

Data collection	No specific code and software were used for data collection.
Data analysis	open source tools:<br>scopetools v0.11 (https://anaconda.org/singleronbio/scopetools)<br>Seurat v2.3<br>InferCNV v1.3.6<br>Monocle v2.0<br>R ggpubr v0.2.5<br>cellphoneDB v2.0<br>GEPIA 1 (http://gepia.cancer-pku.cn/)<br>Slingshot v1.4.0<br>SingleR v1.0.0<br>Harmony v1.0<br>R pheatmap v1.0.12<br>Cytoscape v3.7.2

For manuscripts utilizing custom algorithms or software that are central to the research but not yet described in published literature, software must be made available to editors/reviewers. We strongly encourage code deposition in a community repository (e.g. GitHub). See the Nature Research guidelines for submitting code & software for further information.

Data

Policy information about availability of data

All manuscripts must include a data availability statement. This statement should provide the following information, where applicable:

- Accession codes, unique identifiers, or web links for publicly available datasets
- A list of figures that have associated raw data
- A description of any restrictions on data availability

The raw sequencing data were deposited at Gene Expression Omnibus (GSE148071). The published data used for validation or comparation in this study were retrieved from the NCBI Gene Expression Omnibus database accession code GSE13190712, GSE992549 and ArrayExpress under Accessions E-MTAB-61498. The remaining data are available within the Article, Supplementary Information or available from the authors upon request.

Field-specific reporting

Please select the one below that is the best fit for your research. If you are not sure, read the appropriate sections before making your selection.

- [x] Life sciences
- [ ] Behavioural & social sciences
- [ ] Ecological, evolutionary & environmental sciences

For a reference copy of the document with all sections, see nature.com/documents/nr-reporting-summary-flat.pdf

Life sciences study design

All studies must disclose on these points even when the disclosure is negative.

Sample size	Sample size for scRNA-seq was determined by the availability of patient samples.<br>No statistical tests were performed for sample size calculation.<br>The exact number of samples used for each figure is informed in each legend or manuscript description.
Data exclusions	All criteria for data exclusion were pre-established.<br><br>We removed cells that had either lower than 200 or higher than 5,000 expressed genes. Furthermore, we discarded cells with more than 30,000 UMIs and mitochondria content higher than 30%.<br><br>Within each lineage, we applied an iterative process to remove putative doublet clusters, if any, and re-clustered the remaining cells. Putative doublets were identified by double positive expressions of the canonical marker genes of all major cell types.<br><br>We excluded patients with unknown status of mutation profiles, when comparing between patient subtypes.
Replication	Immunohistochemical (IHC) staining of CD15 and LOX-1 were performed for three patients and replicated for 3 times each.
Randomization	All 42 late-stage, unresectable NSCLC patients were recruited randomly in this study.
Blinding	Blinding to the origin of biopsy samples was not impossible. There was no specific blinding grouping applied in this research. All analysis were performed under selectively grouping and conditions.

Reporting for specific materials, systems and methods

We require information from authors about some types of materials, experimental systems and methods used in many studies. Here, indicate whether each material, system or method listed is relevant to your study. If you are not sure if a list item applies to your research, read the appropriate section before selecting a response.

Materials & experimental systems

n/a	Involved in the study	
x		Antibodies
x		Eukaryotic cell lines
x		Palaeontology
x		Animals and other organisms
	x	Human research participants
x		Clinical data

Methods

n/a	Involved in the study	
x		ChIP-seq
x		Flow cytometry
x		MRI-based neuroimaging

Human research participants

Policy information about studies involving human research participants

Population characteristics	Population characteristics were summarized in Figure 1A, Supplementary Table 1 and Extended Supplementary Data.
Recruitment	Participants were recruited randomly from November 2018 to August 2019.<br><br>Previous single cell studies of lung cancer were mainly focus on early-staged NSCLC patients. The late-stage landscape of lung cancer especially lung squamous carcinoma was mostly absent. A Biased recruitement for late-stage, unresectable NSCLC patients were done in this research.
Ethics oversight	The study was approved by the Ethical Committee of Shanghai Pulmonary Hospital (K18-089-1).

Note that full information on the approval of the study protocol must also be provided in the manuscript.
